# Genetic regulatory axis between AGR2 and ESR1 promotes breast cancer progression

**DOI:** 10.1371/journal.pone.0351873

**Published:** 2026-07-01

**Authors:** M. Aiman Mohtar, Delphine Fessart, Chia Yuh Cai, Syazalina Zahari, Saiful Effendi Syafruddin, Muhammad-Redha Abdullah-Zawawi, Valerie Le Morvan, Said Taouji, Ted Hupp

**Affiliations:** 1 UKM Medical Molecular Biology Institute (UMBI), Universiti Kebangsaan Malaysia, Jalan Yaacob Latiff, Kuala Lumpur, Malaysia; 2 ARTiSt Group, University of Bordeaux, INSERM U1312, Institut Bergonie, 229 Cours de l’Argonne, Bordeaux, France; 3 Institute of Genetics and Cancer, MRC Institute of Genetics & Molecular Medicine, University of Edinburgh, Edinburgh, Scotland, United Kingdom; Dr Vishwanath Karad MIT World Peace University, INDIA

## Abstract

Anterior gradient protein 2 (AGR2), a member of the protein disulfide isomerase family, plays a critical role in endoplasmic reticulum proteostasis and has been implicated in breast cancer progression. However, the downstream regulatory programs and signaling pathways governed by AGR2 remain incompletely defined. Here, we employed CRISPR–Cas9-mediated knockout of AGR2 in breast cancer cells to systematically investigate the functional and transcriptional consequences of AGR2 loss. AGR2 depletion resulted in significant suppression of cell migration, invasion, and chemoresistance. Unbiased transcriptomic profiling by RNA sequencing revealed extensive differential gene expression, implicating AGR2 in receptor-mediated signaling, oxidative stress responses, and cell adhesion pathways. Protein–protein interaction network analysis identified several highly connected hub genes within the AGR2-regulated transcriptome, including estrogen receptor alpha (ESR1/ERα), cadherin 1 (CDH1), androgen receptor (AR), lymphocyte cell-specific protein-tyrosine kinase (LCK), S100 calcium binding protein P (S100P), parkin RBR E3 ubiquitin ligase (PRKN), and decay-accelerating factor (CD55). Notably, ERα emerged as a prominent node within this network, consistent with prior reports linking ERα and AGR2 biology. Integration with publicly available epigenomic datasets further supports a potential regulatory connection between ERα-associated chromatin landscapes and AGR2 expression. Together, these findings define AGR2-dependent transcriptional networks in breast cancer and identify ESR1-associated signaling as a key pathway perturbed upon AGR2 loss, providing a foundation for future mechanistic studies targeting this regulatory interaction.

## Introduction

Breast cancer is a highly heterogeneous disease encompassing diverse molecular subtypes with distinct clinical behaviors and therapeutic responses [1]. It is the most frequently diagnosed and life-threatening malignancy affecting women and plays a leading role in cancer-related mortality worldwide [2]. The prevalence of breast cancer varies geographically, with higher rates in developed countries, likely due to factors such as lifestyle changes, increased life expectancy, and improved screening programs [3]. Based on global statistics data in 2020, breast cancer secured the top spot in terms of incidence with 2.26 million new cases worldwide and ranking fourth in terms of mortality [4]. Breast cancer consists of four main subtypes that are classified based on the expression of hormone receptors, including estrogen receptor (ER), progesterone receptor (PR), and human epidermal growth factor 2 (HER2) receptor, each with distinct biological characteristics and treatment approaches [5]. The treatment strategy for breast cancer typically involves a combination of surgery, radiation therapy, chemotherapy, targeted therapy, and hormone therapy tailored to the individual patient’s tumor subtype and stage. Encouragingly, localized breast cancer diagnoses exhibit a favorable prognosis, with a five-year survival rate exceeding 80% [6]. While metastatic breast cancer is relatively rare at initial diagnosis, accounting for approximately 7% of cases, a worrisome trend reveals that nearly 30% of patients initially diagnosed with the early-stage disease will ultimately develop recurrent or metastatic breast cancer [7,8]. Despite remarkable advances and breakthroughs in breast cancer research, our understanding of this complex and heterogeneous disease remains incomplete, necessitating further exploration and investigation.

Anterior gradient protein 2 (AGR2) has emerged as a clinically relevant oncogenic factor in breast cancer [9,10]. Originally identified as an estrogen-responsive gene ER–positive breast cancer cells, AGR2 expression has been consistently associated with tumor growth, survival, and resistance to endocrine therapy [11,12]. Early mechanistic studies demonstrated that AGR2 promotes breast cancer cell proliferation and survival through modulation of cyclin D1, estrogen receptor alpha (ESR1), and survivin expression, highlighting its central role in ER-associated oncogenic signaling [13]. AGR2 belongs to the protein disulfide isomerase (PDI) family and contains a thioredoxin-like fold that supports protein folding, maturation, and quality control within the endoplasmic reticulum [14–17]. In epithelial tissues, AGR2 contributes to endoplasmic reticulum proteostasis and differentiation, whereas in cancer, its upregulation is thought to support the elevated protein synthesis and metabolic demands characteristic of malignant cells. In epithelial tissues, AGR2 contributes to endoplasmic reticulum proteostasis and differentiation, whereas in cancer, its upregulation is thought to support the elevated protein synthesis and metabolic demands characteristic of malignant cells [14,18,19].

Beyond its intracellular role (iAGR2), accumulating evidence indicates that AGR2 can also be secreted and function extracellularly (eAGR2), where it has been implicated in tumor–microenvironment interactions, receptor signaling, and epithelial–mesenchymal transition [20–23]. Importantly, several of these oncogenic functions of AGR2 have been reported in estrogen receptor–low or estrogen receptor–negative contexts, indicating that AGR2 can exert tumor-promoting effects independently of classical estrogen signaling [13,24–26]. Despite these observations, the mechanisms governing AGR2 secretion and the broader transcriptional programs regulated by AGR2 across distinct hormonal contexts remain incompletely understood. Although AGR2 was originally characterized as an estrogen-responsive gene and has been extensively studied in ER-positive breast cancer, much of the existing literature has focused on upstream hormonal regulation rather than the downstream consequences of AGR2 loss. Comprehensive analyses by Salmans et al. established AGR2 as a downstream effector of estrogen signaling and proposed its utility as both a biomarker and potential therapeutic target in ER-positive breast cancer [27]. However, emerging evidence suggests that AGR2 also plays ER-independent roles in cancer progression, including regulation of cell survival, stress responses, and invasive behavior in non-luminal breast cancer and other tumor types. Consequently, the extent to which AGR2 governs shared versus context-specific transcriptional networks particularly in metastatic and hormone-independent settings remains poorly defined.

In this study, we employed CRISPR–Cas9-mediated genome editing to generate AGR2-deficient breast cancer cells and combined this approach with transcriptomic profiling to characterize the functional and molecular consequences of AGR2 loss. Through unbiased RNA sequencing and network-based analyses, we identified key pathways and regulatory nodes perturbed following AGR2 depletion, including components of estrogen receptor–associated signaling. Notably, estrogen receptor alpha (ESR1) which encodes for ERα emerged as a prominent hub within the AGR2-regulated transcriptional network, consistent with prior reports linking AGR2 and ER signaling. Together, our findings define AGR2-dependent transcriptional landscapes in breast cancer and provide a framework for future mechanistic studies aimed at dissecting AGR2-associated regulatory interactions in endocrine-responsive disease.

## Materials and methods

*Antibodies and reagents*. All chemicals and solvents were obtained from commercial sources and of high purity or HPLC spectral grade. AGR2 monoclonal antibody 6C5 (#sc-101211, Santa Cruz Biotechnology) was used at a 1:1000 dilution. ESR1 monoclonal antibody (#sc-8644, Santa Cruz Biotechnology) was used at a 1:1000 dilution, HER2 antibody (#2242, Cell Signaling Technology) was used at 1:1000 dilution, eIF2α antibody (#2103, Cell Signaling Technology) was used at a 1:1000 dilution. Phospho-eIF2α (#3597, Cell Signaling Technology) was used at a 1:1000 dilution. GAPDH antibody ((#MAB374, Millipore) was used at a 1:5000 dilution and Beta-actin was used at 1:5000 (#sc-69879, Santa Cruz Biotechnology). Secondary antibodies were Horseradish-peroxidase (HRP) conjugated anti-Rabbit IgG (P0217, Dako) at1:1000 and HRP-conjugated anti-Mouse IgG (P0260, Dako) at 1:1000. Hygromycin B solution was purchased from Nacalai Tesque. The drug Doxorubicin HCL (#D1515) was purchased from Sigma Aldrich and Tamoxifen (CAS 10540-29-1) from Santa Cruz Biotechnology.

Cell culture. The T47D human luminal A breast cancer cells (ATCC HTB-133), MCF-7 (ATCC HTB-22), and HEK293T cells were obtained from the American Type Culture Collection (ATCC, Manassas, VA, USA). The 1833-BoM cells stably expressing LentiCas9 Blasticidin were a gift from Dr Sakari Vanharanta. All cell lines were authenticated using short tandem repeat (STR) profiling (S6 File). Briefly, genomic DNA was analyzed across 22 STR loci, including the gender-determining Amelogenin locus and the male-specific DYS391 locus, using the GenePrint® 24 System (Promega). Fragment analysis was carried out on an ABI 3730XL Genetic Analyzer, and data were analyzed using GeneMapper® v5.0 software (Applied Biosystems™). The STR profile matched the reference profile for the 1833-BOM (MDA-BoM-1833) triple negative breast cancer cell line, confirming cell line identity. These cell lines were confirmed to be mycoplasma negative using the e-Myco^TM^ Mycoplasma PCR Detection Kit (Intron, Kirkland, WA, USA). The T47D, MCF-7, 1833-BoM and HEK293T cell lines were maintained in DMEM (Nacalai Tesque), supplemented with 10% FBS (Tico Europe) and 1% penicillin-streptomycin solution (Nacalai Tesque). All cell lines were cultured in 95% humidified incubators with 5% CO_2._ Conditioned media containing AGR2 was collected from either parental 1833-BoM or MCF-7 cells grown in DMEM media supplemented with 10% FBS (Tico Europe) and 1% penicillin-streptomycin solution (Nacalai Tesque) for 24 h from initial seeding at about 50–60% confluency. The cells were then washed with 1 × PBS and replaced with serum-free DMEM. The cells were grown for 24–48 h before the media was collected and filtered using a Minisart NML 0.45 μM syringe filter (Sartorius, Germany). The filtered conditioned media were either used directly or stored at −80°C.

*Western blot.* Cells were lysed in urea buffer (7 M urea, 0.1 m DTT, 0.1% Triton X-100, 25 mM NaCl, 20 mM HEPES–KOH pH 7.6, 5 mM NaF, 2 mM Na_3_VO_4_, 2.5 mM Na_4_P_2_O_7_). Proteins were quantified using the BCA Assay (Thermo Fisher Scientific). Proteins were separated using 12% SDS-PAGE and then transferred onto a 0.2 µM nitrocellulose membrane (GE Healthcare). The membranes were probed with primary antibodies, followed by secondary antibodies conjugated to HRP. Bound antibody was detected by enhanced chemiluminescence (ECL) as a relative light unit. For the detection of secreted AGR2, cells were cultured according to their respected culture medium in supplemented with 10% FBS (Tico Europe) and 1% penicillin-streptomycin solution (Nacalai Tesque) until about ~70−80% confluency. The supernatants were collected and centrifuged at 4,500 × g for 5 minutes. The supernatants were then concentrated using Amicon® Ultra-15 Centrifugal Filter Unit with 3kDa molecular weight cut-off using centrifugation 4,000 x g at 4 °C. The concentrated supernatants (<500 µl) were then lysed with urea buffer and subjected to western blotting. Band intensities were analyzed using densitometry tool in ImageJ.

*Rescue treatment of AGR2-KO cells with conditioned media.* Parental cells were seeded in 6-well plates and allowed to attach for 16 h to achieve ~70–80% confluency the following day. Cells were washed 1× with sterile PBS to remove residual serum components. Conditioned medium was generated by incubating parental cells in low serum (0.5% FBS) medium for 48 h or until they reached ~80% confluency. Following conditioning, conditioned medium was collected and centrifuged at 300 × g, 5 minutes to remove cells, followed by 2,000 × g for 10 minutes to remove debris. The clarified conditioned medium was then passed through a 0.22 μm filter and either used immediately or aliquoted and stored at −80°C. To ensure comparability across batches, AGR2 abundance in conditioned media was quantified using POETIC platform (OncoGyne lab, INSERM U1312, Bordeaux, France) using a no-wash immunoassay read (unpublished protocol) on an EnVision multilabel plate reader (PerkinElmer), according to the platform’s internal standard operating procedures. For rescue experiments with conditioned media, AGR2-KO cells were seeded in 6-well until they reached confluency. The next day (defined as 0 h), growth medium was replaced with treatment media consisting of AGR2-conditioned media, diluted 2:1 with fresh medium. Cells were incubated for (24–96 h) depending on the downstream readouts.

*Reverse transcription (RT)-PCR.* RNA was extracted using the ReliaPrep Cell and Tissue Miniprep Isolation Kit (Promega). One microgram of total RNA was reverse-transcribed to cDNA by the Superscript III Reverse Transcript Kit (Invitrogen). A two-step RT-PCR was used to analyze mRNA expression of the AGR2, ESR1, AR, LCK, CD55, PARK2, CDH1, S100Pand CXCL1. AGR2 and GAPDH primers used were previously described [28]. Primers for ESR1 were predesigned and obtained from PrimeTime qPCR primers by Integrated DNA Technologies (IDT): ESR1 (Hs.PT.58.14846478), AR (Hs.PT.56a.14520219), LCK (Hs.PT.58.40262054), CD55 (Hs.PT.58.38726940), PRKN (Hs.PT.58.40583803), CDH1 (Hs.PT.58.3324071), S100P (Hs.PT.58.20602479), CXCL1 (Hs.PT.58.39039397), ACTB (Hs.PT.39a.22214847). Real-time PCR was carried out in a 10 μL solution containing cDNA, 2 × SYBR Green Mastermix (Applied Biosystems), cDNA synthesis reaction and RNase-free water in presence of primers in a One-Step Plus Real-time PCR system (Applied Biosystems).

*Wound healing assay.* Cells were grown at the same cell densities in 6-well dishes until they reached confluency. Dishes with non-targeting control (NTC) cultures, AGR2 KO1 and AGR2 KO2 cells were scraped in a straight line with a sterile 200 µl plastic pipette tip, after which the wound healing gap was monitored. Photographs were taken at each time point indicated by phase-contrast light microscopy at 100 × original magnification. The surface area of the gaps was analyzed by ImageJ software, and the wound closure rates were calculated by Prism-GraphPad software.

*MTT assay.* Cells were grown at 5,000 cells/well in a 96-well clear plate and grown to reach optimal population densities within 48–72 h. Spent media were discarded before adding, 50 μL of serum-free media and 50 μL of MTT Reagent (R&D Systems) into each well. Background control wells: 50 μL MTT Reagent and 50 μL cell culture media (no cells) was also included. The plates were incubated at 37°C for 3 h. After incubation, the MTT reagent-supplemented media was removed and 150 μL of MTT solvent was added into each well before the plate was wrapped in foil and incubated at 25°C for 24 h. The plate was shaken on a shaker for 15 minutes before measuring the absorbance. The absorbance for each sample was then read at OD 590 nm, and graphs were plotted to measure the percentage viability of cells.

*Invasion assay.* Cells were starved for 18 h in serum-free DMEM. 1 × PBS was used to wash the cells twice and then the PBS was removed. Cells were then trypsinized by 1 × Trypsin-EDTA 0.5% (Nacalai Tesque) warmed to 37^o^C in a water bath beforehand, and the cells were returned to the incubator until they were detached. Warmed serum-free DMEM containing 5% BSA with Mg^2+^ and Ca^2+^ (Quenching Medium) was added and then pelleted at 2,000 RPM for 5 minutes. Cells were counted to bring to a volume of 6.0 x 10^5^ cells/ml. The assay was performed according to the protocol described in the CHEMICON QCM^TM^ 24-well Collagen-based Invasion Assay Kit (Cat#ECM551, Merck Millipore). The invaded cells on the bottom of the Boyden chamber insert are incubated with Cell Stain Solution (included in the kit). The chamber was washed with sterile water, and the non-invading layer was removed. Invaded cells were viewed under phase contrast light microscopy at 100 × original magnification and photographs were taken. Subsequently, the cells were extracted and transferred onto a microplate reader and a colorimetric measurement was taken on a standard microplate reader at OD 560 nm.

*Cell growth assay*. For each cell lines, cells were seeded in 96-well plates at 3000 cells/well using the Perkin Elmer Janus Mini liquid handling system on POETIC platform as previously described [28]. Briefly, plates were incubated at the indicated time, and medium was changed every day by fresh medium or MCF-7 conditioned medium, which secrete eAGR2. High-content images were acquired at each time point with the Cytation 3 automated microscope (Biotek) at 4 × magnification, and analysis was performed using the Analysis software (Biotek).

*Cloning*. The complementary single-guide RNA targeting AGR2 (sgAGR2) and non-targeting control (sgNTC_1) were designed using CRISPick (https://portals.broadinstitute.org/gppx/crispick/public). The sgRNA sequence used in AGR2 KO1(sgAGR2_1) and KO2 (sgAGR2_2) is 5’-GGAAACTTACAACCAGATTG-3’ (Exon 5) and 5’-AGAGATACCACAGTCAAACC-3’ (Exon 2), respectively. The sequence for sgNTC_1 is 5’-GAGTGTCGTCGTTGCTCCTA-3’. The top and bottom complementary sgRNA oligos were purchased separately (Integrated DNA Technologies) and were designed to harbor *BbsI* restriction overhangs at their respective 5′ and 3′ ends for ligation into *BbsI*-digested pKLV-U6-gRNA(*BbsI*)-PGKHygro2AeGFP plasmid as previously described [29,30]. The top and bottom strand oligos were annealed and subjected to 5′ end phosphorylation using T4 polynucleotide kinase (New England Biolabs). Ligation was performed using T4 Ligase (New England Biolabs) at 16°C for 16 h, and the ligated plasmid was transformed into the chemically competent DH5α *E. coli* strain (New England Biolabs). The presence of the sgAGR2 construct within the expression plasmid was verified by Sanger sequencing. The stables clones MCF7-KO-ESR1 clone 10 and clone 17 were designed and produced by the CRISP’edit platform (Bordeaux, France) using sgRNA sequence 5’-GCTCCGCAAATGCTACGAAG (Exon 3). Assessment of the spectrum and frequency of targeted mutations generated in cell pools were analyzed using Deconvolution of Complex DNA Repair (DECODR) [31].

*Lentiviral production and transduction*. HEK293T cells, seeded on the 6-well plate, were co-transfected with the mixture of packaging plasmids psPAX2 (1.3 μg) and pMD2.G (0.5 μg), and the plasmid of interest (1.5 μg) using Attractene transfection reagent (Qiagen, Germany). The medium containing the lentiviral particles was collected 72 h post-transfection and filtered through a Minisart NML 0.45 μM syringe filter (Sartorius, Germany), followed by storage at −80 °C or used for transduction. For transduction, the media containing the lentiviral particles was added into the cells in the presence of 8 µg/mL polybrene (Merck Millipore, USA). The positively transduced cells were selected with 700 µg/mL Hygromycin B solution (Nacalai Tesque).

*RNA-sequencing*. The samples were prepared according to the MGIEasy RNA Directional library preparation guide with the Dynabeads® mRNA purification kit for mRNA enrichment. The libraries that passed QC were pooled based on library concentration and target data volume for sequencing on the MGI platform, DNBSEQ-G400 set at 100 million total raw reads. The mRNA sample is enrichment was performed with Dynabeads® mRNA Purification Kit before being subjected to RNA fragmentation. The fragmented mRNA is reverse transcribed to cDNA and is followed by a second strand synthesis, end-repair and A-tailing, adapter ligation, and PCR amplification generating 381, 375 and 398 bp library size for NTC, KO1 and KO2, respectively. The amplified library then goes through a denaturation step, circularization, and digestion for subsequent sequencing on the DNBSEQ-G400 sequencer.

*Mapping and identification of differentially expressed genes*. To analyze the expression of genes, RNA-seq raw reads were subjected to Trim Galore v0.6.7 for quality control and trimming. The paired-end reads were trimmed using Cutadapt v3.5.0, followed by quality inspection of clean reads using FastQC v0.11.8. Low-quality reads below the Phred score of 20 were discarded from the original data, and MultiQC v1.14 was used to compile a single report of cleaned reads. In this study, STAR aligner v2.7.10a was employed to align the clean reads to the human reference genome (GRCh38.p13), which was obtained from the Gencode human database (https://www.gencodegenes.org/human/). The chimeric reads were detected by STAR with the parameters ‘–chimSegmentMin and –chimOutType WithinBAM’, and were discarded to ensure the reliability of the sequencing data by removing fragments of RNA sequences that may arise from errors during the sequencing process. The aligned RNA-seq reads were subjected to featureCounts v2.0 to quantify the gene expression levels. Differential gene expression analysis was performed using EdgeR with the parameters; mean read counts > 10, p-adjusted value <0.05 and log_2_FC > 1 and <−1.

*Gene ontology and KEGG pathway enrichment analysis.* Enrichment analyses of Gene Ontology (GO) and the Kyoto Encyclopedia of Genes and Genomes (KEGG) pathways were conducted using ClusterProfiler to investigate the biological functions associated with the DEGs. GO enrichment analysis was categorized into three ontology domains: biological process (BP), molecular function (MF), and cellular component (CC). The parameters used for GO and pathway enrichment were as follows: minimum number of DEGs (minGSSize) = 3, maximum number of DEGs (maxGSSize) = 800, showCategory = 10, and pvalueCutoff = 0.05. For GO enrichment analysis, the DEGs were enriched using the ‘org.HS.e.g.,db’ Bioconductor package, whereas the ‘kegg_organism’ parameter was set to ‘hsa’ to specifically enrich the DEGs to KEGG human pathways.

*Protein-protein interaction network analysis and hubs detection.* The PPI network was constructed to evaluate the relationship among the DEGs after AGR2 knockout. The PPI bulk data was downloaded from HIPPIE v2.3 (https://cbdm-01.zdv.uni-mainz.de/~mschaefer/hippie). HIPPIE is a comprehensive database that integrates protein interactions from various sources, and the reliability of the interactions is supported by confidence scores ranging from 0 to 1, where the cut-off range for the most reliable interactions is 0.73 to 1.0. The PPI bulk data comprising 19,679 proteins and 831,933 interactions (accessed on 29^th^ March 2023) were visualized using Cytoscape v3.9.1. To construct the AGR2-knockout PPI network, the DEGs with the log_2_FC > 1 and <−1 were mapped onto the existing network map, and all the individual nodes that did not interact with any DEGs were dismissed. To gain deeper insights into the impact of AGR2 knockout and its DEG-encoded neighboring proteins, we specifically focused on the down-regulated PPIs. CytoHubba was employed to predict the important DEGs by predicting the top 10 highly connected genes (also called hub genes) in AGR2-knockout PPI using the Maximal Clique Centrality (MCC) method. Further, the relationship between drugs and the top 10 hub genes was analysed by employing the drug-target interactions (DTIs) network datasets obtained from BioSNAP (https://snap.stanford.edu/biodata/datasets/10002/10002-ChG-Miner.html). DTIs offer an opportunity for drug repurposing strategies. The DTIs were filtered based on the Food and Drug Administration (FDA) approved drugs that target the top 10 hub genes and visualized using Cytoscape v3.9.1 software.

*Databases.* TCGA (The Cancer Genome Atlas) was accessed through the cBioPortal for Cancer Genomics: https://www.cbioportal.org (accessed on 25^th^ March 2024). The Genotype Tissue Expression (GTEx was assessed through the GTEx Portal: https://gtexprotal.org (accessed on 25^th^ March 2024). We used a pipeline that processes and unifies RNA-seq data from different studies using Gene Expression Profiling Interactive Analysis 2 [32]. Using the pipeline, we have processed data from the GTEx and TCGA and have corrected for study-specific biases, allowing comparative analysis across studies. The Cancer Cell Line Encyclopedia (CCLE) datasets were downloaded from DepMap portal (https://depmap.org/portal/) (accessed on the 10^th^ December 2023). ChIP-seq datasets were downloaded from ENCODE project database (https://www.encodeproject.org/) (accessed on 12^th^ December 2023). Histone ChIP-seq in MCF-7 (ENCSR752UOD) and TF ChIP-seq in MCF-7 (ENCSR463GOT) were downloaded and viewed using Integrative Genomics Viewer (IGV) v2.18.4.

*Statistical analysis.* Statistical analyses were performed using GraphPad Prism v10 (GraphPad Software, USA) and R (version 4.2.3) unless otherwise stated. Data are presented as mean ± standard error of the mean (SEM) from at least three independent biological replicates, unless specified otherwise. Normality of data distribution was assessed using the Shapiro–Wilk test. For comparisons between two groups, two-tailed unpaired Student’s t-tests were applied for normally distributed data, while non-parametric Mann–Whitney tests were used when normality assumptions were not met. For experiments involving multiple groups or conditions, one-way or two-way ANOVA were performed. For RNA-sequencing analysis, differential gene expression was determined using edgeR, with raw counts normalized and modeled using a negative binomial distribution. Statistical significance was assessed using Benjamini–Hochberg false discovery rate (FDR) correction, with adjusted p-values < 0.05 considered significant. Biological replicates refer to independently generated cell populations or experiments, while technical replicates were averaged prior to statistical testing. Exact statistical tests, sample sizes, and significance thresholds are indicated in the corresponding figure legends.

## Results

**Fig 1:**
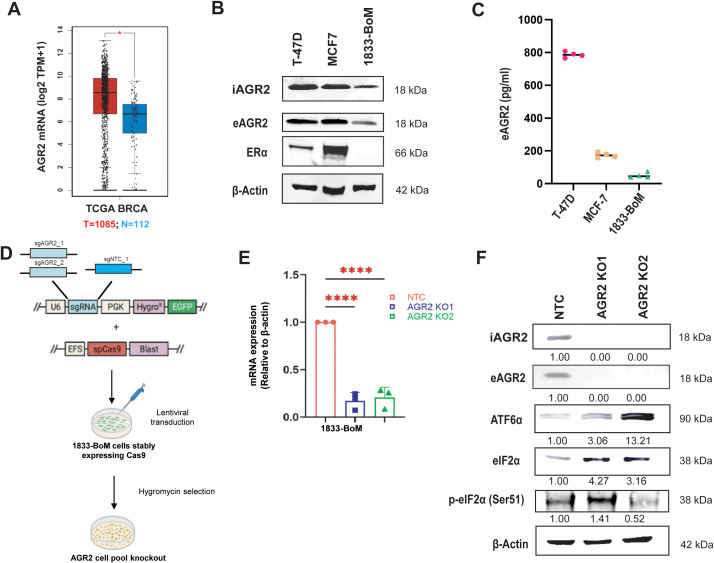
Utilizing CRISPR-Cas9 genome editing tool to generate AGR2 cell pool knockout. **(A)** Box plot comparing mRNA expression of AGR2 in normal (N) and tumour (T) tissues extracted from TCGA-BRCA and matched normal data from GTEx repository **(B)** Expression of intracellular AGR2 (iAGR2) and the extracellular AGR2 (eAGR2) protein released in culture medium as detected by Western blot in a panel of breast cancer cell lines: MCF-7, T47D, and 1833-BOM. The expression of ESR1 also were examined, confirming the positive expression of ESR1 in luminal breast cancer cell lines MCF-7 and T47D, but negative for TNBC cell line 1833-BOM. **(C)** Quantification of eAGR2 in culture medium as detected by an immunoassay. **(D)** Strategy to target AGR2 using CRISPR-Cas9 genome editing tool. Two sgRNA targeting AGR2 and 1 non-targeting controls (NTC) were designed and lentivirally delivered into 1833-BoM cells. **(E)** Pools of lentivirally transduced CRISPR-Cas9 knockout of 1833-BoM cells were subjected to real-time PCR showing relative mRNA level of AGR2 expression. Data are presented as mean ± SEM from triplicate measurements across three independent experiments. *p < 0.05, **p < 0.01, ***p < 0.001, ns (non-significant), one-way ANOVA. **(F)** Pools of lentivirally transduced CRISPR-Cas9 knockout of 1833-BoM cells were subjected to Western blotting using AGR2 and a subset of UPR antibodies (ATF6α, eIF2α, and phosphorylated-eIF2α (Ser51). Band intensities were quantified using densitometry tool and were normalized against that of B-actin and the ratios are indicated below each band.

*CRISPR-mediated AGR2 targeting affects cancer cell phenotypes.* Gene-centric expression analysis using publicly available RNA sequencing datasets from The Cancer Genome Atlas (TCGA) and Genotype-Tissue Expression (GTEx) cohorts revealed that AGR2 mRNA is significantly upregulated in breast cancer tissues compared to normal breast tissues (Fig 1A). To complement this transcriptomic analysis at the protein level, we investigated AGR2 expression in both intracellular (iAGR2; from whole cell lysate) and extracellular (eAGR2; culture media) fractions of three different breast cancer cell lines: MCF7, T47D, and 1833-BoM. Immunoblotting confirmed that the iAGR2 protein was expressed in each breast cancer cell lines, as detected in the whole cell lysate (Fig 1B). Notably, we also detected the presence of eAGR2 at the molecular weight size as iAGR2 in the culture media, confirming that AGR2 is actively secreted by these breast cancer cells using western blot (Fig 1B) and immunoassay (Fig 1C). These findings are consistent with previous reports suggesting AGR2 functions as both an intracellular signaling protein and a secreted factor involved in tumor progression. To further investigate the functional role of AGR2 in breast cancer and identify potential modulators of its signaling network, we employed a CRISPR/Cas9-mediated loss-of-function approach. The bone metastatic 1833-BoM cell line, was selected as our model due to its moderate AGR2 expression, making it suitable for loss-of-function studies. Importantly, this model also enabled assessment of AGR2 function independently of estrogen receptor–driven signaling, thereby allowing interrogation of AGR2-mediated effects across broader breast cancer contexts, including metastatic disease [14,18,24].

For precise genome editing, we designed two single guide RNAs (sgRNAs) targeting AGR2. Non-targeting control (NTC) cells, transduced with an sgRNA lacking a genomic target, were used as negative controls (Fig 1D). Lentiviral delivery of CRISPR/Cas9 components resulted in efficient genome editing, as confirmed by Sanger sequencing and Deconvolution of Complex DNA Repair (DECODR) analysis, which verified the presence of insertion-deletion (indel) mutations at the expected loci (S1 Fig in S2 File). Immunoblot analysis further confirmed that both sgRNAs effectively reduced iAGR2 and eAGR2 at mRNA (Fig 1E) protein levels (Fig 1F). We designated these cell models as AGR2 KO1 and AGR2 KO2, for subsequent functional assays. To assess CRISPR specificity with respect to the closely related paralog AGR3, we examined AGR3 mRNA expression following CRISPR-mediated AGR2 knockout. The sgRNAs used in this study were designed specifically against the AGR2 locus and selected to avoid predicted targeting of AGR3. Quantitative RT-PCR analysis showed that AGR3 expression was significantly increased in both AGR2 KO1 and AGR2 KO2 cells (S2 Fig in S2 File). This finding suggests that AGR3 upregulation represents a compensatory transcriptional response to AGR2 depletion rather than unintended CRISPR targeting.

Previous studies have implicated AGR2 in maintaining endoplasmic reticulum homeostasis and regulating the unfolded protein response (UPR) [33]. To determine whether AGR2 loss induces ER stress, we examined the expression of key UPR markers in AGR2-deficient cells. Immunoblot analysis revealed increased levels of ATF6α and total eIF2α in both AGR2 KO1 and KO2 cells compared with non-targeting control cells, indicating perturbation of ER homeostasis following AGR2 depletion. We next assessed phosphorylation of eIF2α at Ser51, a central marker of activation of the PERK–eIF2α arm of the UPR. Notably, phospho-eIF2α levels were differentially regulated between independent AGR2 knockout clones: AGR2 KO1 exhibited increased eIF2α Ser51 phosphorylation, whereas AGR2 KO2 displayed reduced phospho-eIF2α despite elevated total eIF2α levels (Fig 1F). In contrast, ATF6α expression was increased in both AGR2 KO clones, with a more pronounced induction observed in AGR2 KO2 cells. Collectively, these data indicate that loss of AGR2 disrupts ER homeostasis and engages the UPR, but that individual AGR2-deficient clones adopt distinct adaptive responses characterized by differential activation of specific UPR branches. This heterogeneity underscores the context-dependent nature of UPR signaling following AGR2 depletion in breast cancer cells.

**Fig 2:**
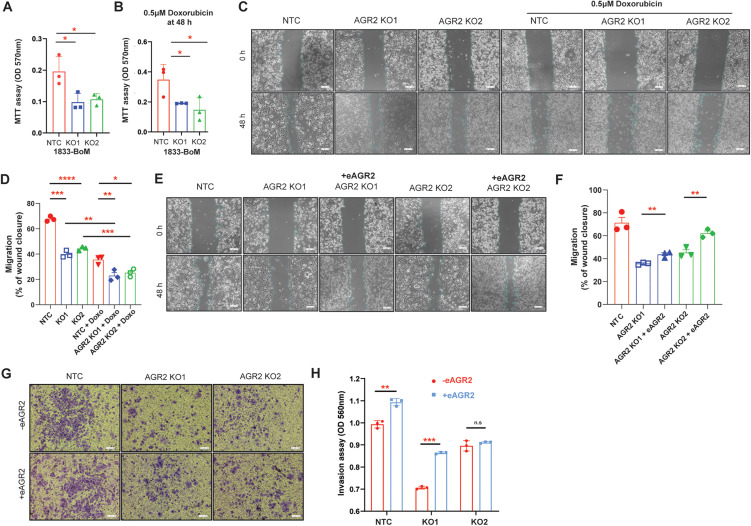
AGR2 supports the growth and motility of breast cancer cells. **(A)** Bar-graphs quantifying cells viability using MTT assay in AGR2 targeted 1833-BOM cells after 48 h of growth. **(B)** Bar-graphs quantifying cells viability using MTT assay after exposure of 0.5uM Doxorubicin for 48 h in AGR2 targeted cells. **(C)** Representative images of wound area covered by the NTC and AGR2-targeted 1833-BOM after wound scratch represented by the reduction of cell-free area, after 48-h treatment with 0.5 μM Doxorubicin in AGR2-targeted cells. **(D)** Bar-graphs showing the percentage of wound closure represented by the reduction of cell-free area, after 48-h treatment with 0.5 μM Doxorubicin in AGR2-targeted cells from **(C)**. **(E)** Representative images of wound area covered by the NTC and AGR2-targeted 1833-BOM after wound scratch at indicated time in the presence or absence of conditioned media containing eAGR2 (+eAGR2) or not. **(F)** Bar-graphs showing the percentage of wound closure represented by the reduction of cell-free area **(E)**. **(G)** Representative images of cell invasion assay performed using Boyden chambers coated with Matrigel, in the presence or absence of +eAGR2. Invaded cells on the lower surface were fixed and stained with crystal violet. **(H)** Bar-graphs showing the quantification of stained invaded cells from (G) by measuring absorbance at OD_560nm_. All data are presented as mean ± SEM from triplicate measurements across three independent experiments. *p < 0.05, **p < 0.01, ***p < 0.001, ns (non-significant), one-way ANOVA, except for Fig 2H (student’s t test).

To evaluate the functional consequences of AGR2 depletion, we performed a series of cell-based phenotypic assays. First, we assessed the proliferation ability of AGR2 KO cells compared to NTC. The results demonstrated that loss of AGR2 significantly impaired cell proliferation (Fig 2A). Next, we investigated whether AGR2 depletion affects the response to chemotherapeutic treatment. Given that previous studies have implicated AGR2 in mediating doxorubicin resistance in breast cancer cells [34,35], we evaluated the sensitivity of AGR2-KO cells to doxorubicin treatment in MTT and wound healing assays (Fig 2B–2D). AGR2-deficient cells exhibited significantly increased susceptibility to doxorubicin-induced cytotoxicity, indicating that AGR2 contributes to chemoresistance. Notably, a rescue experiment using conditioned media from AGR2-overexpressing cells restored both the migratory and wound-healing capacity of AGR2 KO cells (Fig 2E–2F). Given the inherently aggressive phenotype of the 1833-BoM cell line, we further examined the effect of AGR2 depletion on invasive capacity. Upon supplementation with conditioned medium containing extracellular AGR2 (eAGR2), AGR2 KO1 showed a significant restoration of invasive capacity, whereas AGR2 KO2 displayed only a partial and more variable response (Fig 2G–2H). These findings indicate that secreted AGR2 contributes to the invasive phenotype of 1833-BoM cells, although the magnitude of rescue appears to be clone-dependent.

**Fig 3:**
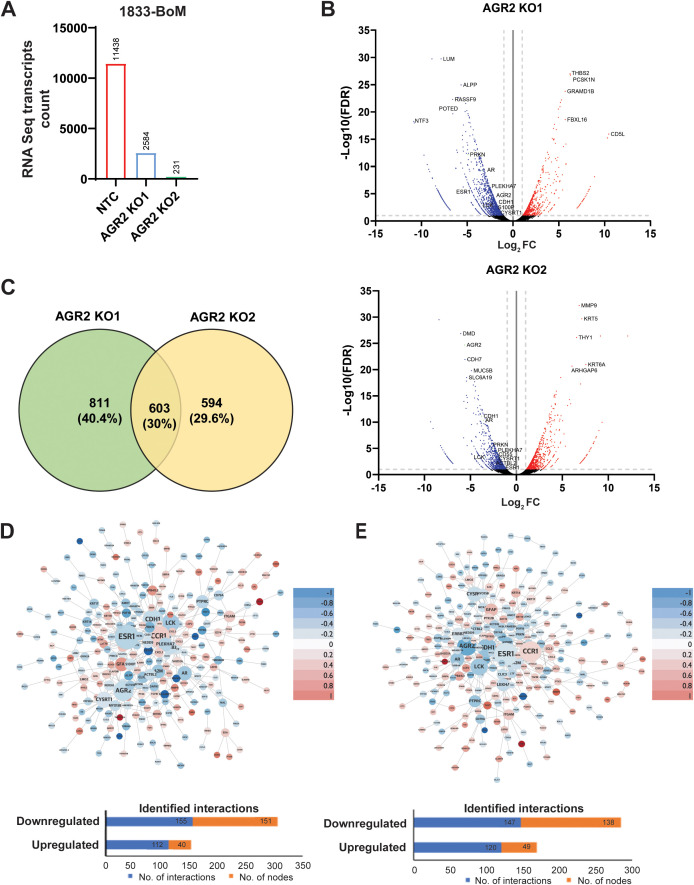
RNA-seq analysis reveals altered transcriptome network in AGR2-targeted breast cancer cells. **(A)** Bar-graphs showing RNA-seq transcript counts of AGR2 in NTC, AGR2 KO1, and AGR2 KO2 cells. **(B)** Volcano plot displaying DEGs in AGR2 KO1 (upper panel) and AGR2 KO2 (lower panel) compared to NTC cells. DEGs were defined as those with adjusted p-value <0.05 and log_2_FC > 1 and <−1. **(C)** Venn diagram showing common DEGs between AGR2 KO1 and AGR2 KO2 groups. **(D-E)** PPI interaction network analysis highlighting downregulated (blue nodes) and upregulated (red nodes) genes in AGR2 KO1 (left panel) and AGR2 KO2 (right panel) groups, along with the corresponding number of identified interactions identified from (bottom panel).

*Transcriptomic profiling reveals AGR2-dependent transcriptional programs.* To define the global transcriptional consequences of AGR2 loss, we performed high-throughput RNA sequencing (RNA-seq) on total RNA extracted from whole-cell lysates of two independent AGR2 knockout clones (AGR2 KO1 and AGR2 KO2) and non-targeting control (NTC) cells. Sequencing generated more than 100 million reads per sample, providing deep coverage for robust differential expression analysis. Consistent with successful genome editing, AGR2 transcript counts were among the most significantly downregulated genes in both AGR2 KO clones compared with NTC cells (Fig 3A), in agreement with the marked reduction in AGR2 protein levels observed by immunoblotting (Fig 1E). These results validate the efficiency of CRISPR-mediated AGR2 targeting and support the reliability of the transcriptomic dataset. Unbiased differential gene expression analysis identified 1,414 differentially expressed genes (DEGs) in AGR2 KO1 and 1,197 DEGs in AGR2 KO2 relative to NTC cells (p < 0.005, |log2 fold change| ≥ 1; Fig 3B and S1 Table). In AGR2 KO1, 412 genes were upregulated and 1002 genes were downregulated, while in AGR2 KO2, 671 genes were upregulated and 526 genes were downregulated, indicating widespread transcriptional remodeling following AGR2 depletion. Comparison of both datasets revealed 603 DEGs commonly altered in the two AGR2 knockout clones (Fig 3C), corresponding to approximately 42.6% of DEGs in AGR2 KO1 and 50.4% of DEGs in AGR2 KO2. We considered these shared genes to represent the most robust core AGR2-dependent transcriptional signature. The remaining non-overlapping DEGs likely reflect a combination of clone-specific transcriptional adaptation, differences in editing outcome, and biological variability between independently derived knockout populations, rather than contradictory responses to AGR2 loss. Notably, interrogation of this AGR2-dependent transcriptome revealed differential expression of several genes encoding previously reported AGR2-interacting or client proteins, including MUC2, MUC5B, MUC5AC, TFF1, TFF3, and FOXA1 [26,36]. These proteins have been shown in prior studies to physically associate, co-expressed with AGR2 or depend on AGR2 for proper folding, secretion, or functional regulation within the secretory pathway. The coordinated transcriptional perturbation of these AGR2-associated factors following AGR2 knockout suggests that loss of AGR2 impacts not only downstream signaling pathways but also broader interaction networks linked to AGR2 function. This observation further supports the utility of CRISPR-based transcriptomic profiling as a complementary approach for uncovering AGR2-associated regulatory and interaction networks beyond direct protein–protein interaction assays.

To minimize the influence of clone-specific transcriptional changes and potential off-target effects, all downstream analyses in this study were restricted to the 603 differentially expressed genes (DEGs) shared between AGR2 KO1 and AGR2 KO2 clones. This common DEG set served as the basis for pathway enrichment analysis, protein–protein interaction (PPI) network construction, and hub-gene identification, ensuring that subsequent findings reflect reproducible AGR2-dependent effects rather than clone-specific perturbations. To further contextualize the AGR2-dependent transcriptional signature identified in this study, we compared our common DEG dataset with a previously published RNA-seq analysis of AGR2-regulated genes in A549 lung cancer cells reported by Martisova et al. [37]. This comparison revealed 72 genes commonly deregulated between the two studies (S3 Fig in S2 File and S1 Table). While the overall overlap was partial, likely reflecting differences in cancer type, cellular context, and AGR2 targeting strategy, several conserved AGR2-associated genes were identified.

Construction of protein–protein interaction (PPI) networks using the commonly deregulated DEGs resulted in PPI network comprises of 498 nodes and 365 edges, indicating a high degree of interconnectivity (Fig 3D–3E). Strong overlap between the AGR2 KO1 and KO2 networks further demonstrated the reproducibility of the transcriptional perturbations induced by AGR2 loss. Gene ontology (GO) and Kyoto Encyclopedia of Genes and Genomes (KEGG) pathway enrichment analyses of the common DEGs revealed significant suppression of biological processes related to receptor-mediated signaling, oxidative stress responses, cell adhesion, and vesicle trafficking (S4 Fig in S2 File). These pathways are consistent with known roles of AGR2 in intracellular signaling and cellular homeostasis.

**Fig 4:**
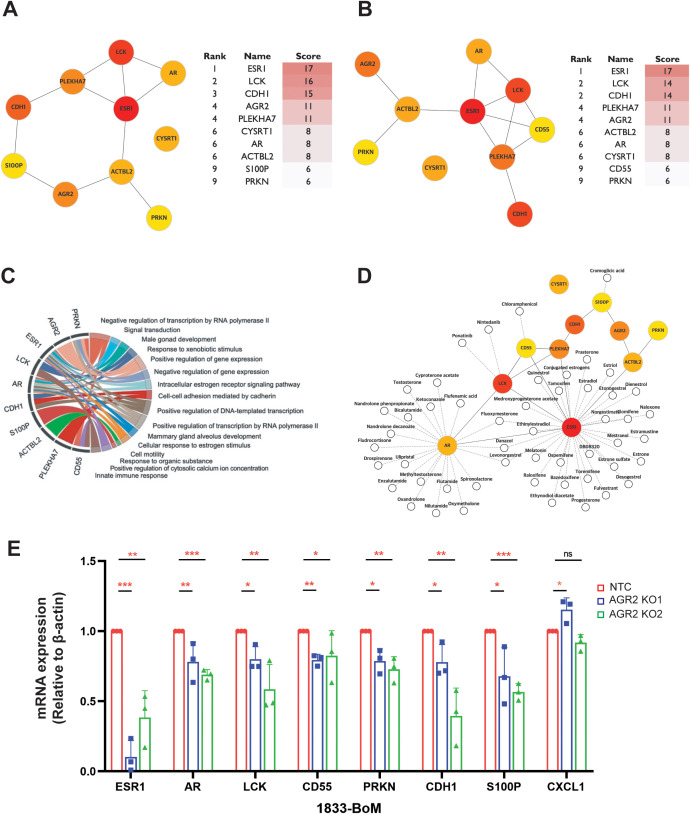
Identification of targetable hub genes as potential modulators in the AGR2 signaling axis. **(A-B)** Top 10 hub genes connected to AGR2 identified through protein–protein interaction (PPI) network analysis in AGR2 KO1 and AGR2 KO2, respectively. **(C)** Chord diagram showing the association between significantly enriched pathways (right) and the combined top 10 hub genes identified from AGR2 KO groups (left). **(D)** Gene-drug interaction analysis revealing FDA-approved drugs that potentially target the identified hub genes within AGR2-associated network. **(E)** Validation of mRNA levels of representative genes from RNA-seq, hub genes, and cross-study comparison analyses by RT-PCR. Data are presented as mean ± SEM from triplicate measurements * *p* < 0.05; ** *p* < 0.01; *** *p* < 0.001; **** *p* < 0.0001; ns (non-significant), one-way ANOVA.

To prioritize functionally relevant regulatory nodes from the AGR2-dependent transcriptome, we implemented a network-based target selection strategy designed to refine large-scale RNA-seq data into biologically meaningful candidates. Specifically, network topology analysis was performed on downregulated differentially expressed genes (DEGs) shared between both AGR2 knockout clones, thereby focusing on reproducible AGR2-dependent effects and minimizing clone-specific or off-target signals. Highly interconnected genes (hub genes), defined by degree and centrality metrics, were selected as candidate regulators most likely to exert functional influence within the AGR2-associated network. This analysis yielded a highly consistent set of top-ranked hub genes across both AGR2 knockout clones, including ESR1, androgen receptor (AR), lymphocyte cell-specific protein-tyrosine kinase (LCK), cadherin 1 (CDH1), S100 calcium binding protein P (S100P), parkin RBR E3 ubiquitin ligase (PRKN), pleckstrin homology domain containing A7 (PLEKHA7), actin beta-like 2 (ACTBL2), and complement decay-accelerating factor (CD55) (Fig 4A–4B). The recurrence of these hub genes across independent AGR2 knockout models supports their role as central components of AGR2-regulated transcriptional programs, rather than stochastic or clone-specific changes.

To assess the clinical relevance of these prioritized candidates, we examined their expression relationships using publicly available TCGA breast cancer RNA-seq datasets. AGR2 expression showed significant positive correlations with all identified hub genes except CD55, while LCK exhibited an inverse correlation (S5 Fig in S2 File), indicating coordinated regulation of these nodes in human tumors. Functional enrichment analysis further demonstrated that the hub genes converge on biological processes central to breast cancer progression, including ERα–associated signaling, transcriptional regulation, cell motility, cell adhesion, and innate immune responses (Fig 4C). These findings suggest that AGR2 influences interconnected signaling pathways rather than isolated downstream targets.

Given their centrality within the AGR2-regulated network and their involvement in clinically relevant pathways, we next evaluated the therapeutic tractability of the hub genes. Drug–gene interaction analysis using FDA-approved and investigational compound databases identified 51 candidate compounds targeting five hub genes (ESR1, AR, LCK, CD55, and S100P), with ESR1 emerging as the most druggable node, based on the number of predicted interacting compounds (Fig 4D). Extended protein–protein interaction analysis further revealed interacting partners that may represent secondary targets or enable combinatorial therapeutic strategies within the AGR2 signaling axis. Finally, to experimentally validate this multi-layer prioritization approach, we performed quantitative RT-PCR on a representative subset of hub genes (ESR1, AR, LCK, PRKN, CDH1, S100P and CD55) using independently prepared AGR2 knockout and non-targeting control samples. Consistent with the RNA-seq data, all tested genes exhibited significant downregulation in AGR2-deficient cells relative to controls (Fig 4E), confirming the robustness of the transcriptomic analysis and validating the effectiveness of this network-based strategy in identifying functionally relevant AGR2-associated targets.

We next performed a cross-study comparison with the RNA-seq dataset reported by Martisova et al. in A549 lung cancer cells to identify recurrent AGR2-responsive genes across independent cellular contexts [35]. This analysis revealed a subset of overlapping genes, including S100P and CDH1, which were also part of our downregulated hub-gene set and were validated by RT-PCR in the present study. We additionally examined CXCL1, another overlapping gene identified in the Martisova dataset. Unlike the hub genes prioritized from our downregulated PPI network, CXCL1 showed a clone-dependent response following AGR2 loss, with significant upregulation observed in AGR2 KO1 but not AGR2 KO2 (Fig 4E). This finding suggests that AGR2 perturbation can induce both suppressive and context-dependent compensatory transcriptional responses, depending on the gene and cellular background. Thus, while the primary PPI analysis was intentionally focused on shared downregulated genes to define a core AGR2-dependent hub network, the dataset by Martisova comparison provides an orthogonal layer of validation by highlighting recurrently AGR2-responsive genes across models, even when the magnitude or direction of response is not fully conserved.

*Experimental validation of AGR2-ERα regulatory axis.* The network-based prioritization of AGR2-dependent transcriptional programs identified ESR1 as a top-ranked hub gene, recurrent across independent AGR2 knockout clones and central within clinically relevant signaling pathways. While the regulatory relationship between ERα and AGR2 has been previously reported in ER–positive breast cancer, the emergence of ERα as a key node from an unbiased, AGR2-centric loss-of-function analysis provided a strong rationale to revisit this interaction in the context of our dataset. Importantly, this finding suggests that ERα is not merely an upstream hormonal regulator of AGR2, but may also represent a core component of AGR2-associated transcriptional networks perturbed upon AGR2 loss.

**Fig 5:**
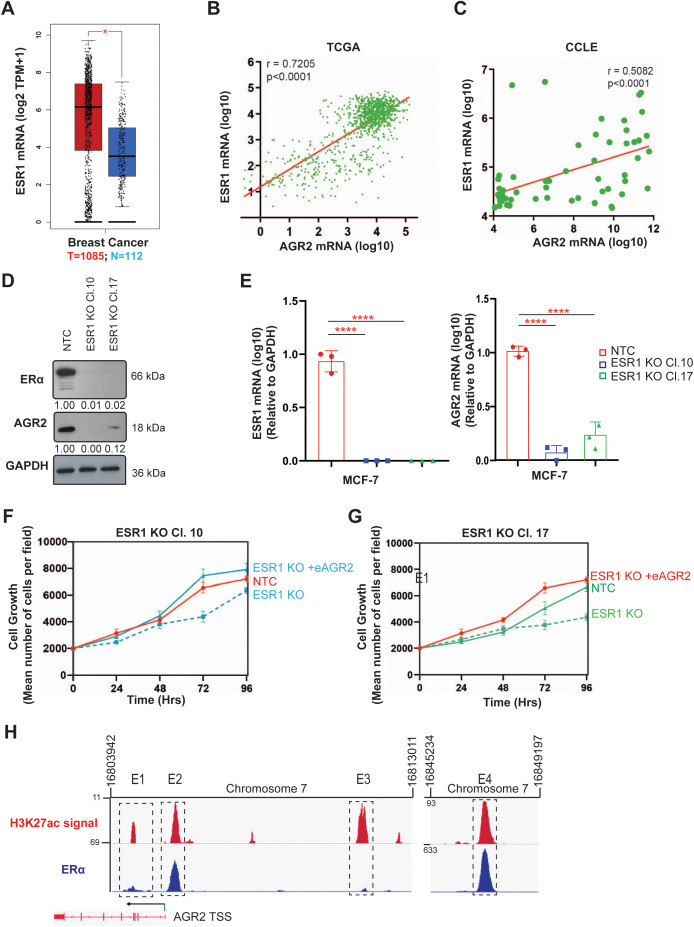
ESR1 drives the expression of AGR2 in breast cancer cells. **(A)** Box plot comparing mRNA expression of ESR1 in normal (N) and tumour (T) tissues extracted from TCGA-BRCA and matched normal data from GTEx repository. **(B-C)** Pearson correlation analysis between AGR2 expression and ER**α** protein levels in breast cancer patient tissues from the TCGA dataset (B) and cell lines from the CCLE dataset **(C)**. Each dot represents an individual patient or cell line. Correlation was measured by Pearson’s correlation test. **(D)** Western blot analysis of AGR2 and ER**α** protein levels in MCF7-NTC as compared to MCF7 depleted in ESR1 (ESR1-KO clone 10 & clone 17). GAPDH was used as a loading control. Band intensities were quantified using densitometry tool and were normalized against that of GAPDH and the ratios are indicated below each band. **(E)** Relative mRNA level of AGR2 and ESR1 expression in MCF-7 cells as determined by RT-PCR. GAPDH was used as a housekeeping gene. **(F-G)** Growth curves of MCF7 NTC and ESR1 KO cells (clone 10 in G, clone 17 in H) grown in 96-well plates, in the presence or absence of extracellular AGR2 (+eAGR2) in the conditioned medium. Cell proliferation was assessed by measuring cell number per field area. Data are presented as mean ± SEM from at least three independent experiments. Two-way ANOVA with Time (Row factor) and Treatment (Column factor) as fixed effects. P-values were calculated as Interaction (Row Factor x Column Factor); p < 0.01 for Clone 10 and p < 0.0001 for Clone 17. **(H)** Integrative Genomics Viewer (IGV) track showing ChIP-seq enrichment of ESR1 and H3K27ac near the AGR2 locus spanning 53-kilobases relative to the AGR2 transcription start site (TSS) in MCF7 cells, based on ENCODE data. The dotted boxes highlight potential four enhancer-like regulatory elements in labeled as elements 1-4 (E1-E4).

Consistent with this observation, RNA-seq analysis of AGR2 knockout cells revealed significant disruption of ER–associated signaling pathways, further implicating AGR2 in the modulation of ER-driven transcriptional programs (S6 Fig in S2 File). To contextualize this relationship at the population level, we performed comparative analyses using publicly available TCGA breast cancer RNA-seq datasets, which confirmed robust ESR1 gene expression in breast cancer tissues (Fig 5A). Pearson correlation analyses of both the TCGA cohort (Fig 5B) and the Cancer Cell Line Encyclopedia (CCLE) dataset (Fig 5C) demonstrated a significant positive correlation between AGR2 and ESR1 gene expression, supporting coordinated regulation of these genes in breast cancer.

To experimentally validate the AGR2–ERα relationship in an appropriate estrogen receptor–positive context, we utilized the MCF-7 cell line, a well-established model for ER signaling in breast cancer [38]. CRISPR–Cas9-mediated knockout of ESR1 gene in MCF-7 cells resulted in efficient genome editing, as confirmed by indel formation at the ESR1 locus (S7 Fig in S2 File). Loss of ESR1 led to a marked reduction in AGR2 expression at both the protein (Fig 5D) and mRNA levels (Fig 5E), indicating that AGR2 expression is at least partially dependent on ESR1 activity. To assess the functional consequences of this regulatory interaction, we performed cell proliferation assays comparing ESR1-depleted cells with non-targeting controls under multiple conditions. Notably, supplementation with conditioned media containing extracellular AGR2 (eAGR2) partially restored the proliferative capacity of ESR1 knockout cells (Fig 5F–5G), suggesting that secreted AGR2 can compensate, at least in part, for impaired ERα signaling. These findings support a functional interdependence between AGR2 and ESR1 that extends beyond transcriptional regulation.

Previous studies have demonstrated ERα binding at regulatory regions of the AGR2 locus, including ERα ChIP-seq peaks associated with estrogen-responsive AGR2 expression [27]. we sought to refine the regulatory landscape of AGR2 by integrating transcription factor occupancy with chromatin activation states. To this end, we analyzed publicly available ERα ChIP-seq and H3K27ac datasets from the ENCODE consortium [36]. Consistent with prior reports, multiple ESR1 binding sites were detected within the AGR2 promoter and upstream regulatory regions (Fig 5H). Overlay of ERα occupancy with H3K27ac enrichment revealed that only a subset of ERα-bound regions coincided with active chromatin marks, whereas other ERα peaks lacked detectable H3K27ac signal in the cellular contexts examined. This distinction indicates that ERα binding alone is not sufficient to define transcriptionally active regulatory elements and suggests context-dependent enhancer utilization at the AGR2 locus. Based on the overlap between ERα ChIP-seq peaks and H3K27ac enrichment, we annotated four enhancer-like elements (E1–E4) that likely represent the most transcriptionally relevant ERα-associated regions within the AGR2 locus (Fig 5H). These elements represent putative ERα-associated cis-regulatory regions that may contribute to AGR2 transcriptional control. Notably, a prominent ERα-occupied region overlapping with H3K27ac enrichment was observed within a previously reported enhancer interval, nominating this region as a candidate ERα-associated active enhancer linked to AGR2 transcription. While these integrative analyses refine candidate regulatory elements controlling AGR2 expression, direct functional interrogation of individual enhancer sites was not performed in the current study.

## Discussion

Genetic ablation of AGR2 in mouse models has been associated with severe phenotypes, including gastrointestinal dysfunction and premature lethality, underscoring the essential role of AGR2 in epithelial homeostasis and limiting its utility for dissecting AGR2 signaling pathways in vivo [39–42]. As a result, relatively few studies have established isogenic cellular systems to interrogate AGR2-dependent molecular programs. Many prior in-vitro studies have relied on transient siRNA-mediated knockdown or partial suppression of AGR2, rather than complete gene inactivation [43–46]. While siRNA-based approaches are valuable for rapid functional screening, they are inherently limited by incomplete target suppression, variable knockdown efficiency, transient effects, and potential off-target silencing, which can complicate interpretation of downstream phenotypes and molecular networks. In addition, residual AGR2 expression following siRNA treatment may mask AGR2-dependent regulatory programs, particularly for genes or pathways that are sensitive to dosage effects [26]. In this study, we employed CRISPR–Cas9-mediated genome editing to generate AGR2-deficient breast cancer cells using a pooled knockout strategy that preserves cell viability while enabling stable and sustained AGR2 loss. This approach allowed robust phenotypic and transcriptomic analyses while avoiding the limitations associated with transient or partial gene suppression. At the same time, the use of independently derived knockout pools likely captures both a shared AGR2-dependent transcriptional core and clone-specific adaptive responses, which may explain why only a subset of DEGs overlapped between AGR2 KO1 and KO2. Although transcriptomic analyses of AGR2 perturbation have been reported in other cancer models, these studies differ in cellular context, perturbation strategy, and analytical scope [37,47]. Consistent with AGR2’s established oncogenic role, loss of AGR2 in this study resulted in marked impairments in proliferation, migration, and invasion, reinforcing its contribution to cancer cell fitness and metastatic potential. These findings are in line with previous loss-of-function studies across multiple cancer models and validate the relevance of our experimental system [41,48,49]. Interestingly, AGR2 depletion was also accompanied by increased AGR3 expression, suggesting a compensatory response within the AGR family. However, this increase was not sufficient to rescue the phenotypic defects associated with AGR2 loss, supporting a non-redundant role for AGR2 in this setting.

To define AGR2-dependent regulatory networks, we conducted unbiased RNA sequencing using isogenic AGR2 knockout models. This approach enabled systematic identification of transcriptional programs perturbed upon AGR2 loss and facilitated integrative analyses linking differential expression, network topology, and pathway enrichment. By focusing on reproducibly downregulated genes shared between independent AGR2 knockout clones, we minimized clone-specific and off-target effects and prioritized biologically robust AGR2-associated targets. Network-based analyses highlighted a subset of highly interconnected hub genes, including ESR1, AR, LCK, CDH1, S100P, CD55 and PRKN, several of which have established roles in hormone signaling, cell adhesion, and tumor progression. Among these, ESR1 and AR emerged as prominent regulators. AGR2 is a well-established estrogen-responsive gene implicated in ER-positive breast cancer growth and endocrine resistance [9,24,50,51]. Likewise, AGR2 and its paralog AGR3 have been shown to be androgen-responsive in prostate cancer, with AR binding sites identified in AGR2 regulatory regions [52]. However, the transcriptional circuitry governing AGR2 expression remains incompletely defined, particularly across distinct hormonal contexts. The role of androgen signaling in breast cancer is complex, with AR exerting context-dependent oncogenic or tumor-suppressive effects depending on molecular subtype [53]. Notably, AR-positive subsets of triple-negative breast cancer have demonstrated sensitivity to AR-targeted therapies [54]. Given the shared steroidogenic origins of estrogen and androgen signaling [55], our findings position AGR2 as a potential integrator of hormone-associated transcriptional networks across breast cancer subtypes.

Another notable hub identified was LCK, a Src-family tyrosine kinase previously reported as an AGR2 interactor through proximity labeling and affinity purification approaches [23]. LCK is overexpressed in breast cancer and associated with poor clinical outcomes [56] and has been implicated in regulating cancer cell motility through EGFR-dependent uPA secretion [57]. These observations raise the possibility that LCK may also influence AGR2 secretion or downstream signaling, an avenue that warrants further investigation. We also identified CYSRT1 as a highly connected node within the AGR2-regulated network. Given AGR2’s function as a protein disulfide isomerase, this association is mechanistically plausible, although the role of CYSRT1 in breast cancer remains poorly characterized. Collectively, these findings expand the AGR2-associated signaling landscape and identify novel candidates for future functional interrogation [17,58]. Importantly, our drug–gene interaction analysis highlights the therapeutic tractability of this network and supports ongoing efforts to target AGR2-associated oncogenic pathways [59–62].

**Fig 6:**
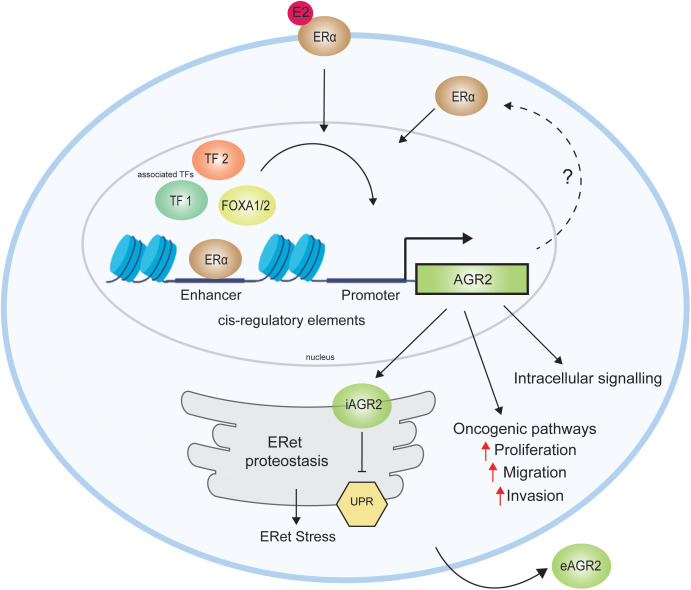
Proposed mechanistic model of the AGR2–ERα regulatory axis in breast cancer. Estrogen (E2)–activated ERα binds cis-regulatory elements at the AGR2 locus, including promoter and enhancer regions, potentially in cooperation with pioneer and associated transcription factors such as FOXA1/2, to promote AGR2 transcription. Intracellular AGR2 (iAGR2) supports endoplasmic reticulum proteostasis and limits unfolded protein response (UPR) activation, whereas secreted AGR2 (eAGR2) is proposed to act via autocrine and paracrine signaling to enhance oncogenic phenotypes, including proliferation, migration, and invasion. Transcriptomic perturbation and functional analyses suggest a potential, context-dependent feedback loop in which AGR2 activity may sustain or reinforce ESR1-associated transcriptional programs, particularly in estrogen receptor–positive settings. Solid arrows indicate interactions supported by experimental or public datasets, whereas dashed arrows denote proposed regulatory relationships requiring further mechanistic validation. ERet, endoplasmic reticulum; TF, transcription factor.

Building on these discovery-driven analyses, we examined the relationship between AGR2 and ERα in greater detail. AGR2 overexpression has been linked to resistance to anti-estrogen therapies such as tamoxifen, and prior studies have suggested bidirectional interactions between AGR2 and ER signaling [12,13]. In our study, AGR2 loss resulted in reduced ESR1 expression, while ESR1 depletion similarly decreased AGR2 expression and extracellular secretion, indicating functional interdependence between these factors. Although earlier work demonstrated reductions in ER protein levels following transient AGR2 silencing [49], a mechanistic framework explaining this relationship has remained elusive. The summary model (Fig 6) integrates our experimental findings with existing genetic epigenetic evidence to propose a regulatory architecture in which ERα directly contributes to AGR2 transcription through engagement of cis-regulatory elements, while AGR2 activity may, in turn, modulate ERα-associated signaling programs in a context-dependent manner. By integrating loss-of-function experiments with analysis of publicly available ChIP-seq datasets, we identified ERα binding at AGR2 regulatory regions co-localized with active chromatin marks, supporting a transcriptional component to this regulatory interaction. Importantly, not all ERα binding sites exhibited features of active enhancers, underscoring the context-specific nature of AGR2 regulation and justifying the depiction of selective enhancer engagement in the proposed model. Future studies should interrogate transcription factor motif enrichment within these candidate enhancer-like elements, including potential motifs for ESR1 and cooperating pioneer factors such as FOXA family members, to better define the cis-regulatory logic governing AGR2 expression. While additional mechanistic studies are required to fully delineate causality, our data support a working hypothesis in which AGR2 and ERα may participate in a potential, reinforcing regulatory loop rather than a constitutive or universal feedback mechanism.

Beyond hormone signaling, AGR2’s role in tumor progression likely extends to interactions with the tumor microenvironment. ERα status has been implicated in immune modulation and therapy resistance in breast cancer [63,64], raising the possibility that the AGR2–ERα axis may influence cytokine signaling or immune evasion pathways [63,64]. Furthermore, although AGR2 is classically localized to the endoplasmic reticulum, its extracellular secretion confers additional pro-tumorigenic functions [20,65]. The mechanisms governing AGR2 secretion, potentially involving non-canonical secretory routes or post-transcriptional regulation, remain poorly understood and represent an important area for future investigation.

In summary, this study defines AGR2-dependent transcriptional networks using a genome editing–enabled, systems-level approach and identifies key regulatory nodes that link AGR2 to hormone signaling, metastatic behavior, and therapeutic vulnerability. By integrating unbiased transcriptomics, network analysis, and targeted validation, our findings extend current understanding of AGR2 biology and highlight the AGR2–ERα axis as a candidate therapeutic target, particularly in endocrine-resistant breast cancer. Future studies dissecting the transcriptional, post-transcriptional, and secretory mechanisms governing AGR2 function will be critical for translating these insights into effective therapeutic strategies.

## Supporting information

S1 Table Supplementary Tables 1. The processed RNA-seq data. (XLSX)

S2 File Supplementary S1–S7 Figs. (PDF)

S3 File Raw western blot images. (PDF)

S4 Table Raw cell-based assay quantification and statistical analyses. (XLSX)

S5 Table Raw qPCR datasets and statistical analyses. (XLSX)

S6 File STR Profiling. (PDF)
