# Supplementary material for: Genetic regulatory axis between AGR2 and ESR1 promotes breast cancer progression
Source: PLoS One. 2026 Jul 1;21(7):e0351873. doi: 10.1371/journal.pone.0351873 (PMC13322506; doi:10.1371/journal.pone.0351873)
Supplement: S2 File — (PDF) [file pone.0351873.s002.pdf]

Supplementary Figs 1-7

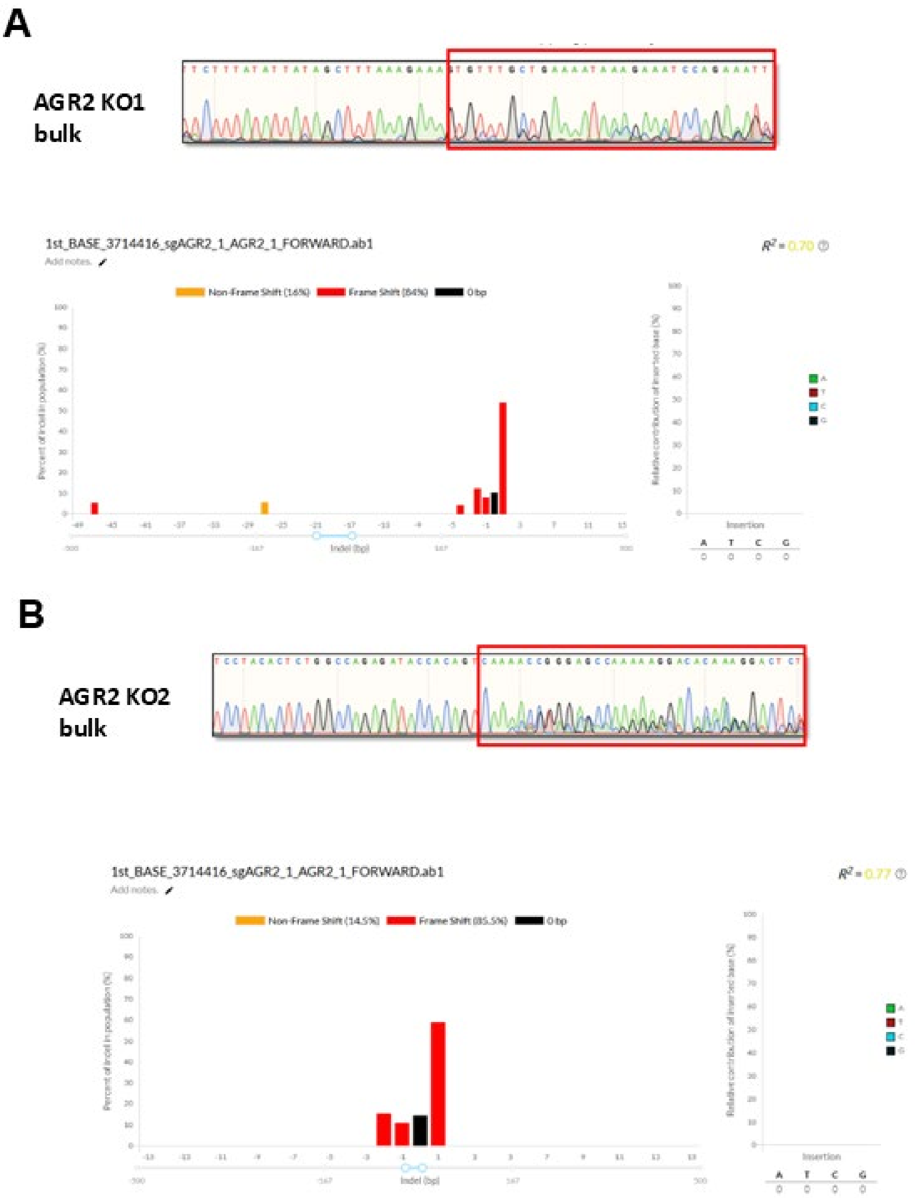

Figure S1. Deconvolution of Complex DNA Repair (DECODR) analysis of AGR2 targeted 1833-BoM cells. DECODR analysis of sgAGR2_1 (A) and sgAGR2_2(B) targeting AGR2 in 1833-BoM. Top panel showing DNA sequencing chromatograms near the Cas9 cleavage site showing overlapping chromatogram peaks and region for deconvolution (red box). Bottom panel showed the indel spectrum in AGR2 target locus.

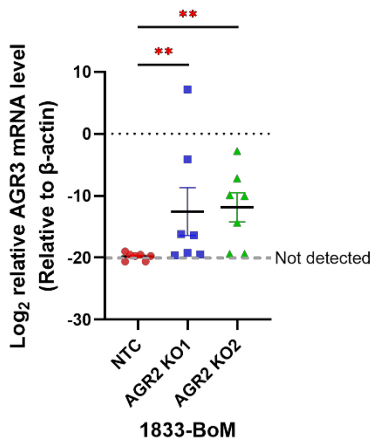

Figure S2. Relative mRNA level of AGR3 expression in 1833-BoM AGR2 KO cells as determined by RT-PCR. GAPDH was used as a housekeeping gene.

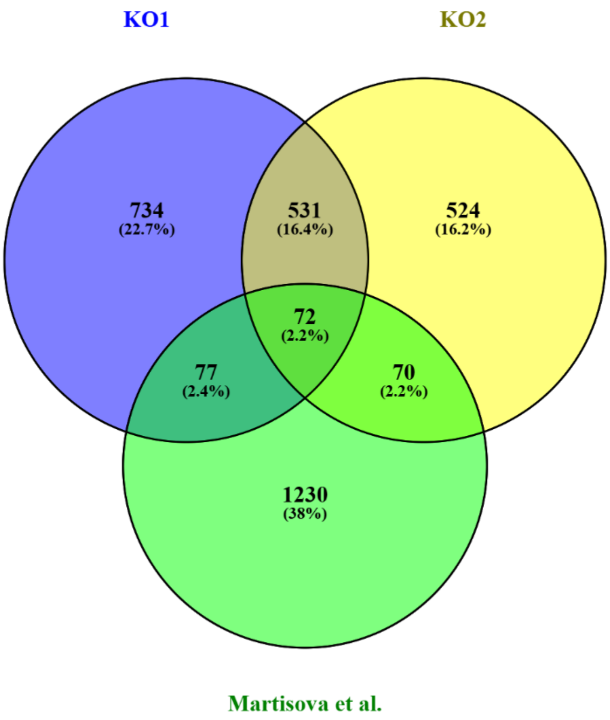

Figure S3. Venn diagram showing common DEGs from RNA-seq experiments between breast cancer cells 1833-BOM AGR2 KO1 and AGR2 KO2 groups from this study and AGR2 knockout in A549 lung cancer cells from Martisova et al (2022)**.** 72 genes were found to be common in both studies.

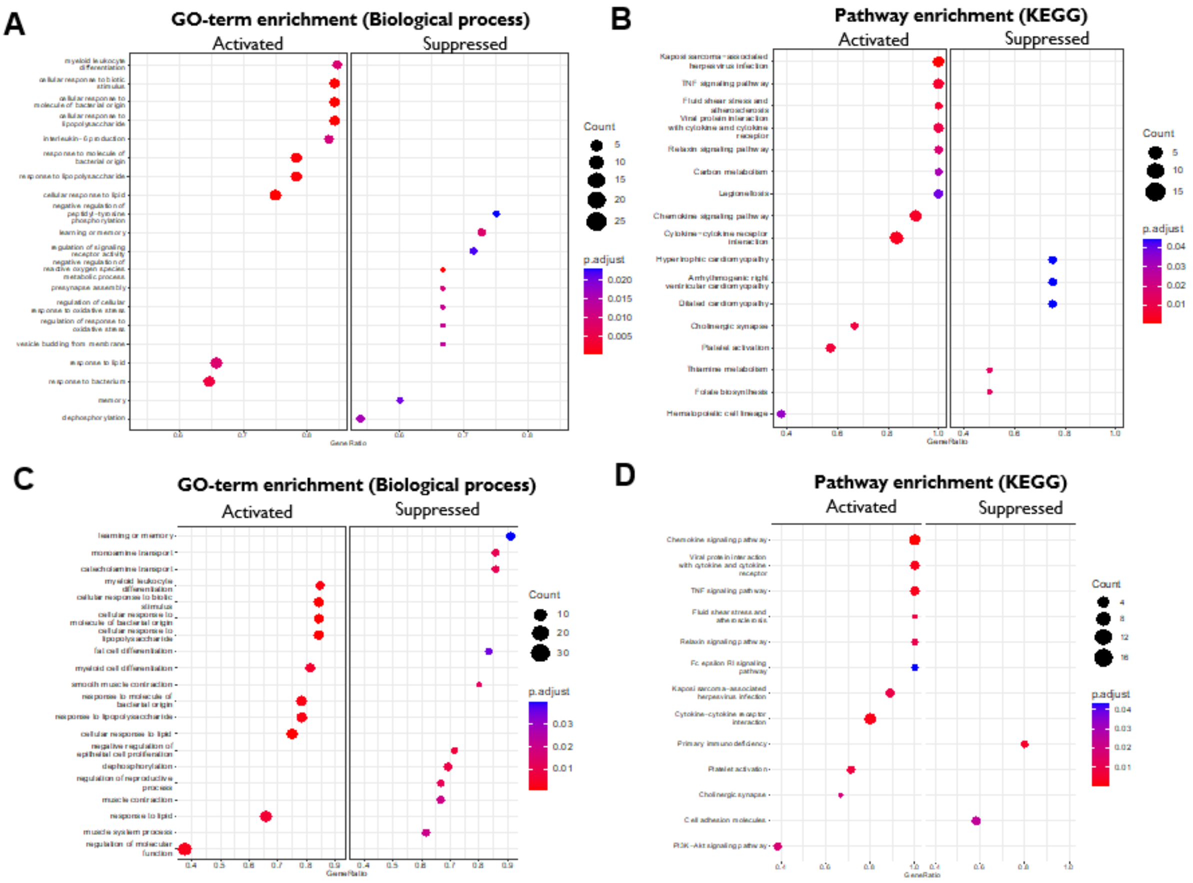

Figure S4. GO-term enrichment based on biological process and KEGG pathway enrichment for (A-B) KO1 and (C-D) KO2.

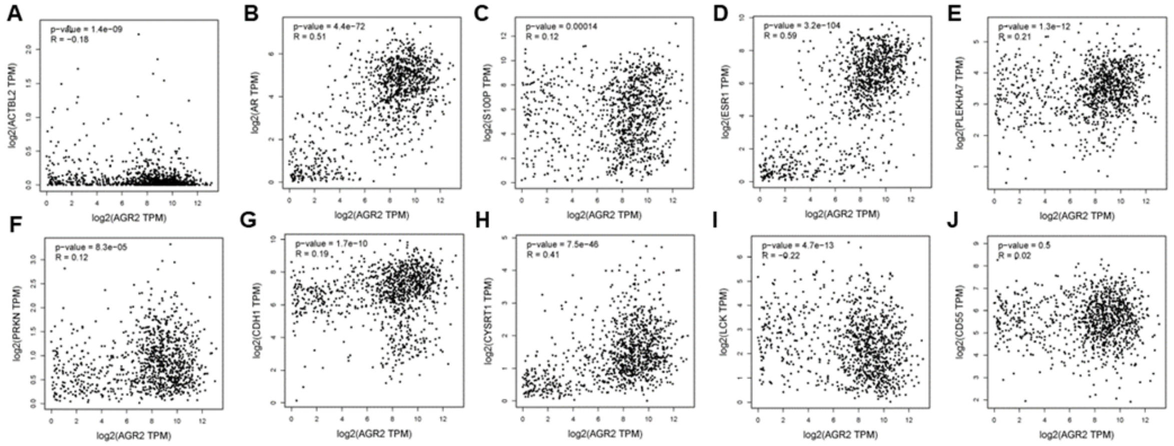

Figure S5. Spearman correlation analysis of AGR2 with the top 10 hub genes in TCGA breast tissues with (A) ACTBL2 (B) AR (C) S100P (D) ESR1 (E) PLEKHA7 (F) PRKN (G) CDH1 (H) CYSRT1 (I) LCK (J) CD55.

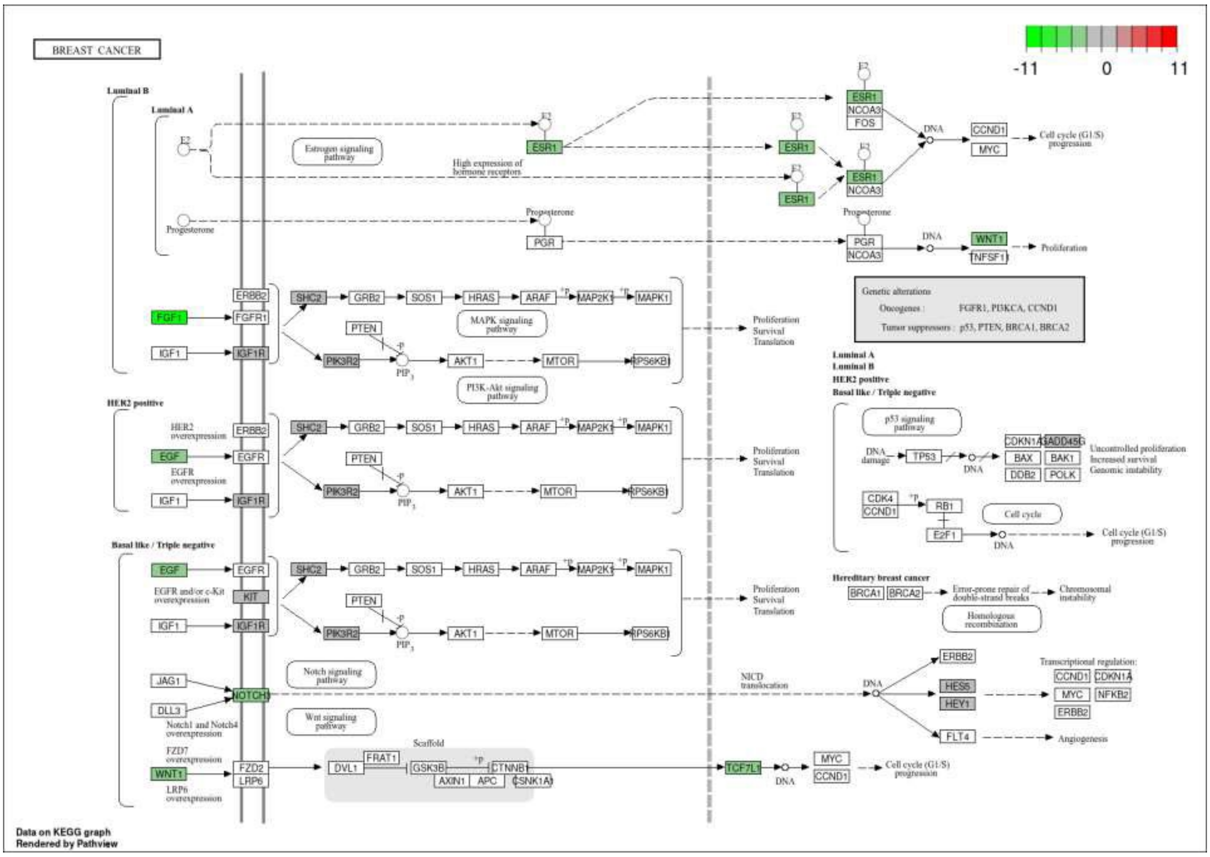

Figure S6. KEGG Pathview from whole differential expressed genes from RNA-Seq for KO1 in Breast Cancer highlighting ESR1 pathway.

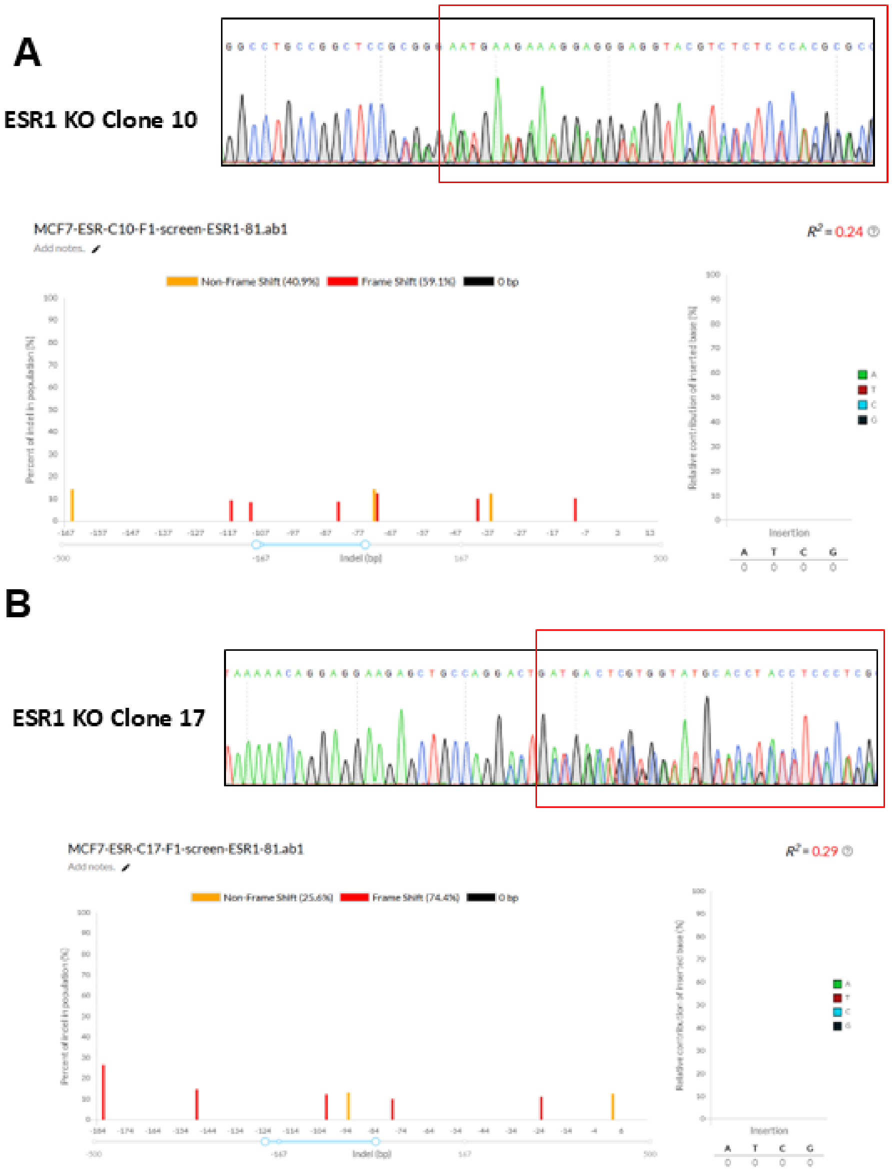

Figure S7. Deconvolution of Complex DNA Repair (DECODR) analysis of ESR1 targeted MCF-7 cells. DECODR analysis of sgESR1 for clone 10 (A) and clone 17 (B) targeting ESR1 in MCF-7 cells. Top panel showing DNA sequencing chromatograms near the Cas9 cleavage site showing overlapping chromatogram peaks and region for deconvolution (red box). Bottom panel showed the indel spectrum in ESR1 target locus.
