# Supplementary material for: Genetic regulatory axis between AGR2 and ESR1 promotes breast cancer progression
Source: PLoS One. 2026 Jul 1;21(7):e0351873. doi: 10.1371/journal.pone.0351873 (PMC13322506; doi:10.1371/journal.pone.0351873)
Supplement: S3 File — (PDF) [file pone.0351873.s003.pdf]

Full western blot scans for Figure 1B

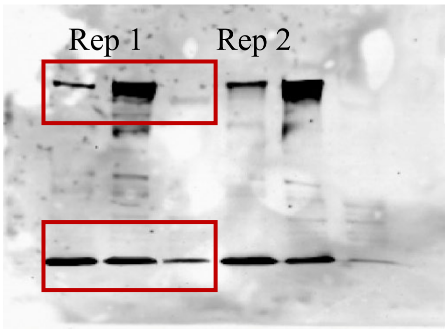

ESR1

iAGR2

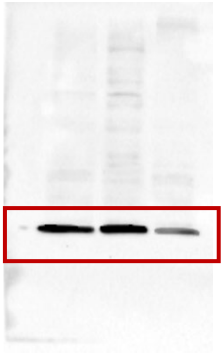

eAGR2

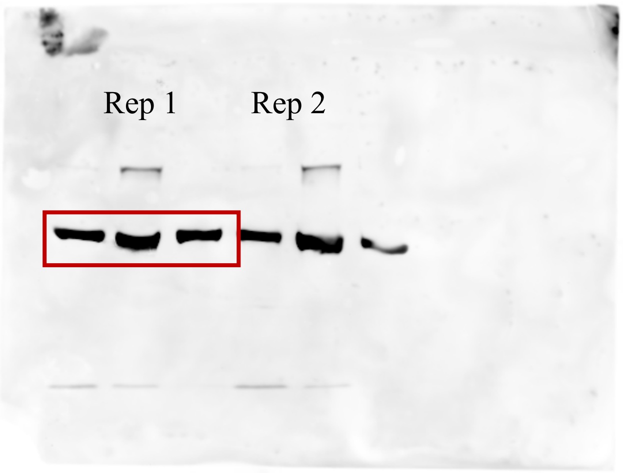

β-actin

Full scans for Figure 1F

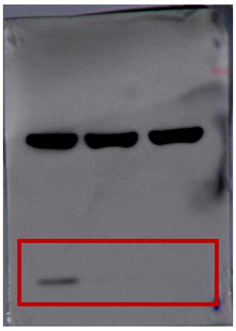

β-actin

iAGR2

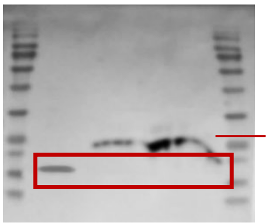

IL6 (not for this study)

eAGR2

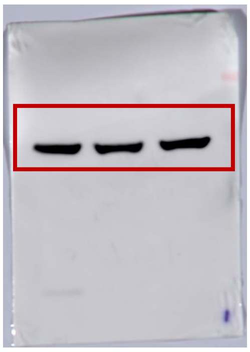

β-actin

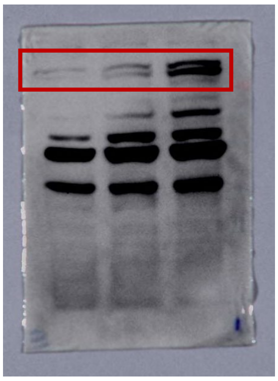

ATF6α

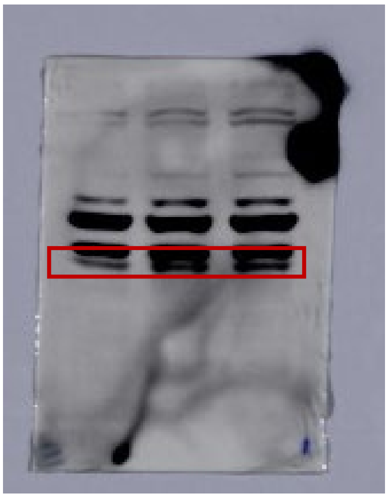

eIF2α

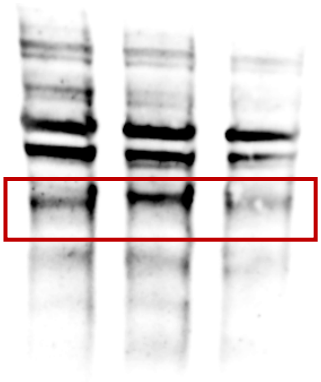

p-eIF2α (ser51)

Full scans for Figure 5D

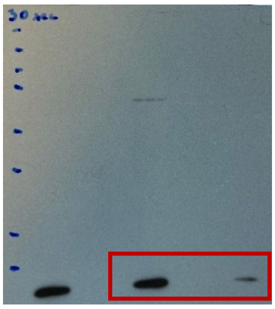

iAGR2

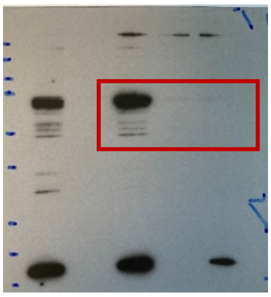

ESR1

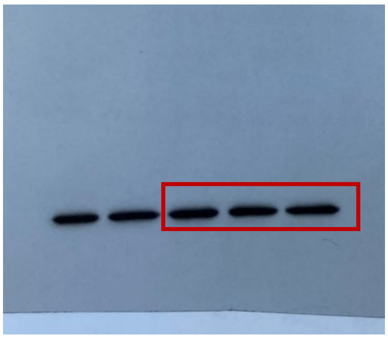

GAPDH
