# Supplementary material for: Genetic regulatory axis between AGR2 and ESR1 promotes breast cancer progression
Source: PLoS One. 2026 Jul 1;21(7):e0351873. doi: 10.1371/journal.pone.0351873 (PMC13322506; doi:10.1371/journal.pone.0351873)
Supplement: S6 File — (PDF) [file pone.0351873.s006.pdf]

Fragment Analysis Services
Human Cell Line Authentication

CUSTOMER DETAILS			
UKM Medical Molecular Biology Institute (UMBI),<br>Jalan Yaacob Latiff, Bandar Tun Razak,<br>56000 Cheras, Kuala Lumpur, Malaysia.<br>Attn : Dr. Mohamad Aimanuddin bin Mohtar/ Nurshahirah Ashikin binti Moidu	**Order ID**	**:**	FA13620
	**Date of Submission**	**:**	9/11/2023
	**Date Completed**	**:**	19/12/2023

1. Sample Information

**Sample Name**	:	1833-BOM
**Cell line designation (Customer provided)**	:	MDA-1833-BOM
**Methodology**	:	Twenty-two short tandem repeat (STR) loci plus the gender determining locus, Amelogenin and male-specific DYS391 locus were amplified using the commercially available GenePrint® 24 System from Promega. The sample was processed using the ABI 3730XL Genetic Analyzer. Data were analyzed using GeneMapper® v5.0 software (Applied Biosystems™). Appropriate positive and negative controls were run and confirmed for each sample submitted.

Note: The ATCC trademark and trade name, any and all ATCC catalog numbers are trademarks of the American Type Culture Collection. PowerPlex is a registered trademark of Promega Corporation. Applied Biosystems, ABI Prism and GeneMapper are registered trademarks of Life Technologies Corporation.

2. STR Profiling Results

STR Locus	Test Sample: 1833-BOM		Reference Sample: MDA-1833-BOM	
Amelogenin	X		X	
D3S1358	16		16	
D1S1656	15	17		
D2S441	14	15		
D10S1248	14	16		
D13S317	13		13	
Penta E	11			
D16S539	12		12	
D18S51	11	16	11	16
D2S1338	20	21	20	21
CSF1PO	12	13	12	13
Penta D	11	14		
TH01	7	9.3	7	9.3
vWA	15		15	
D21S11	30	33.2	30	33.2
D7S820	8		8	
D5S818	12		12	
TPOX	8	9	8	9
DYS391				
D8S1179	13		13	
D12S391	17			
D19S433	12	14.2	11	
FGA	22	23	22	23
D22S1045	16			

Total alleles in the Test Sample (a)	12
Total alleles in the References Sample (b)	12
SHARED alleles, Test and Reference Samples (c)	12
Percent Match for Test and Reference Samples (d)	100%

NOTE:

i. The allele match algorithm compares the loci highlighted in grey only (8 core loci plus Amelogenin).

ii. Loci highlighted in grey (8 core STR loci plus Amelogenin) are used to verify cell identity using STR Database.

iii. Match Algorithm = $\frac{\text{SHARED ALLELES } \mathbf{(c)} \times 2}{\text{TOTAL ALLELES in the Test Sample } \mathbf{(a)} + \text{TOTAL ALLELES in the Reference Sample } \mathbf{(b)}}$

iv. Percent Match **(d)** = Match Algorithm x 100

3. Test Results

Refer (✓)	Comments
✓	The submitted sample profile showed **100% match** with the following reference cell line provided by customer.
	The submitted sample profile showed < 80% match for the ATCC human cell line(s) in the STR database. The submitted sample needs further testing for authentication of relatedness.
	The submitted sample profile is human, but not a match for any profile in the STR database.
	The submitted sample is a mixture. Multiple peaks are observed in fragment analysis results.

4. **Quality Control**
   Passed

5. **Test Laboratory Location**
   APICAL SCIENTIFIC Laboratory
   Apical Scientific Sdn. Bhd.
   No. 17, Jalan BS 7/1C,
   Taman Perindustrian Bukit Serdang,
   Seri Kembangan 43300,
   Selangor, Malaysia.

Report Issued by:
Stanley Lim Soon Hong (Laboratory Officer)

Report Authorized by:
Noor Emira Binti Ahmad Shuhaimi (Laboratory Officer)

This report shall not be reproduced except in full without approval of the laboratory. The result reported relates only to the test item received and tested in-house. The content of this report and any of the attachment is confidential to the recipient only.

End of Report

Fragment Analysis Services
Human Cell Line Authentication

CUSTOMER DETAILS

UKM Medical Molecular Biology Institute (UMBI),<br>Jalan Yaacob Latiff, Bandar Tun Razak,<br>56000 Cheras, Kuala Lumpur, Malaysia.<br>Attn : Dr. M. Aiman Mohtar	**Order ID**	**:**	FA13799
	**Date of Submission**	**:**	9-Mar-26
	**Date Completed**	**:**	13-Mar-26

1. Sample Information

**Sample Name**	:	HEK293T
**Cell line designation**<br>**(Customer provided)**	:	HEK293T Human embryonic kidney cells
**Methodology**	:	Twenty-two short tandem repeat (STR) loci plus the gender determining locus, Amelogenin and male-specific DYS391 locus were amplified using the commercially available GenePrint® 24 System from Promega. The sample was processed using the ABI 3730XL Genetic Analyzer. Data were analyzed using GeneMapper® v5.0 software (Applied Biosystems™). Appropriate positive and negative controls were run and confirmed for each sample submitted.

Note: The ATCC trademark and trade name, any and all ATCC catalog numbers are trademarks of the American Type Culture Collection. PowerPlex is a registered trademark of Promega Corporation. Applied Biosystems, ABI Prism and GeneMapper are registered trademarks of Life Technologies Corporation.

2. STR Profiling Results

STR Locus	Test Sample: HEK293T			Reference Sample: HEK293T Human embryonic kidney cells		
Amelogenin	X			X		
D3S1358	15	16	17	15	16	17
D1S1656	15	17.3		15	17.3	
D2S441	11	15		11	15	
D10S1248	14	15		14		
D13S317	12	14		12	14	
Penta E	7	15		7	15	
D16S539	9	12		9	12	
D18S51	17	18	19	17	18	
D2S1338	18	19		19		
CSF1PO	11	12		11	12	
Penta D	9	10		9	10	
TH01	7	9.3		7	9.3	
vWA	16	19		16	19	
D21S11	28	30.2		28	30.2	
D7S820	11			11		
D5S818	8	9		8	9	
TPOX	11			11		
DYS391						
D8S1179	12	14		12	14	
D12S391	19	21		19	21	
D19S433	18			18		
FGA	23			23		
D22S1045	16			16		

Total alleles in the Test Sample **(a)**	15
Total alleles in the References Sample **(b)**	15
SHARED alleles, Test and Reference Samples **(c)**	15
Percent Match for Test and Reference Samples **(d)**	100%

NOTE:

i. The allele match algorithm compares the loci highlighted in grey only (8 core loci plus Amelogenin).

ii. Loci highlighted in grey (8 core STR loci plus Amelogenin) are used to verify cell identity using STR Database.

iii. Match Algorithm = $\frac{\text{SHARED ALLELES } \mathbf{(c)} \times 2}{\text{TOTAL ALLELES in the Test Sample } \mathbf{(a)} + \text{TOTAL ALLELES in the Reference Sample } \mathbf{(b)}}$

iv. Percent Match **(d)** = Match Algorithm x 100

3. Test Results

Refer (✓)	Comments
✓	The submitted sample profile showed **100% match** with the following reference cell line provided by customer.
	The submitted sample profile showed < 80% match for the ATCC human cell line(s) in the STR database. The submitted sample needs further testing for authentication of relatedness.
	The submitted sample profile is human, but not a match for any profile in the STR database.
	The submitted sample is a mixture. Multiple peaks are observed in fragment analysis results.

4. Quality Control
Passed

5. Test Laboratory Location
APICAL SCIENTIFIC Laboratory
Apical Scientific Sdn. Bhd.
No. 17, Jalan BS 7/1C,
Taman Perindustrian Bukit Serdang,
43300 Seri Kembangan,
Selangor, Malaysia.

Report Issued by:
Che Nurul Fariza Che Hasnan (Laboratory Technician)

Report Authorized by:
Stanley Lim Soon Hong (Laboratory Officer)

This report shall not be reproduced except in full without approval of the laboratory. The result reported relates only to the test item received and tested in-house. The content of this report and any of the attachment is confidential to the recipient only.

End of Report

Fragment Analysis Services
Human Cell Line Authentication

CUSTOMER DETAILS			
UKM Medical Molecular Biology Institute (UMBI),<br>Jalan Yaacob Latiff, Bandar Tun Razak,<br>56000 Cheras, Kuala Lumpur, Malaysia.<br>Attn : Dr. M. Aiman Mohtar	**Order ID**	**:**	FA13799
	**Date of Submission**	**:**	9-Mar-26
	**Date Completed**	**:**	13-Mar-26

1. Sample Information

**Sample Name**	:	MCF-7
**Cell line designation (Customer provided)**	:	MCF-7 breast cancer cells
**Methodology**	:	Twenty-two short tandem repeat (STR) loci plus the gender determining locus, Amelogenin and male-specific DYS391 locus were amplified using the commercially available GenePrint® 24 System from Promega. The sample was processed using the ABI 3730XL Genetic Analyzer. Data were analyzed using GeneMapper® v5.0 software (Applied Biosystems™). Appropriate positive and negative controls were run and confirmed for each sample submitted.

Note: The ATCC trademark and trade name, any and all ATCC catalog numbers are trademarks of the American Type Culture Collection. PowerPlex is a registered trademark of Promega Corporation. Applied Biosystems, ABI Prism and GeneMapper are registered trademarks of Life Technologies Corporation.

2. STR Profiling Results

STR Locus	Test Sample: MCF-7			Reference Sample: MCF-7 breast cancer cells		
Amelogenin	X			X		
D3S1358	16			16		
D1S1656	11	15.3		11	15.3	
D2S441	10	14		10	14	
D10S1248	14			14		
D13S317	11			11		
Penta E	7	12		7	12	
D16S539	11	12		11	12	
D18S51	14			14		
D2S1338	21	23		21	23	
CSF1PO	10			10		
Penta D	12			12		
TH01	6			6		
vWA	14	15		14	15	
D21S11	30			30		
D7S820	8	9		8	9	
D5S818	11	12		11	12	
TPOX	9	12		9	12	
DYS391						
D8S1179	10	14		10	14	
D12S391	18	20		18	20	
D19S433	13.2	14.2		13	14	
FGA	23	24	25	23	24	25
D22S1045	15	16		15	16	

Total alleles in the Test Sample **(a)**	14
Total alleles in the References Sample **(b)**	14
SHARED alleles, Test and Reference Samples **(c)**	14
Percent Match for Test and Reference Samples **(d)**	100%

NOTE:

i. The allele match algorithm compares the loci highlighted in grey only (8 core loci plus Amelogenin).

ii. Loci highlighted in grey (8 core STR loci plus Amelogenin) are used to verify cell identity using STR Database.

iii. Match Algorithm = $\dfrac{\text{SHARED ALLELES } \mathbf{(c)} \times 2}{\text{TOTAL ALLELES in the Test Sample } \mathbf{(a)} + \text{TOTAL ALLELES in the Reference Sample } \mathbf{(b)}}$

iv. Percent Match **(d)** = Match Algorithm x 100

3. Test Results

Refer (✓)	Comments
✓	The submitted sample profile showed **100% match** with the following reference cell line provided by customer.
	The submitted sample profile showed < 80% match for the ATCC human cell line(s) in the STR database. The submitted sample needs further testing for authentication of relatedness.
	The submitted sample profile is human, but not a match for any profile in the STR database.
	The submitted sample is a mixture. Multiple peaks are observed in fragment analysis results.

4. Quality Control
Passed

5. Test Laboratory Location
APICAL SCIENTIFIC Laboratory
Apical Scientific Sdn. Bhd.
No. 17, Jalan BS 7/1C,
Taman Perindustrian Bukit Serdang,
43300 Seri Kembangan,
Selangor, Malaysia.

Report Issued by:
Che Nurul Fariza Che Hasnan (Laboratory Technician)

Report Authorized by:
Stanley Lim Soon Hong (Laboratory Officer)

This report shall not be reproduced except in full without approval of the laboratory. The result reported relates only to the test item received and tested in-house. The content of this report and any of the attachment is confidential to the recipient only.

End of Report

Fragment Analysis Services
Human Cell Line Authentication

CUSTOMER DETAILS

UKM Medical Molecular Biology Institute (UMBI),<br>Jalan Yaacob Latiff, Bandar Tun Razak,<br>56000 Cheras, Kuala Lumpur, Malaysia.<br>Attn : Dr. M. Aiman Mohtar	**Order ID**	**:**	FA13799
	**Date of Submission**	**:**	9-Mar-26
	**Date Completed**	**:**	13-Mar-26

1. Sample Information

**Sample Name**	:	T-47D
**Cell line designation (Customer provided)**	:	T-47D breast cancer cells
**Methodology**	:	Twenty-two short tandem repeat (STR) loci plus the gender determining locus, Amelogenin and male-specific DYS391 locus were amplified using the commercially available GenePrint® 24 System from Promega. The sample was processed using the ABI 3730XL Genetic Analyzer. Data were analyzed using GeneMapper® v5.0 software (Applied Biosystems™). Appropriate positive and negative controls were run and confirmed for each sample submitted.

Note: The ATCC trademark and trade name, any and all ATCC catalog numbers are trademarks of the American Type Culture Collection. PowerPlex is a registered trademark of Promega Corporation. Applied Biosystems, ABI Prism and GeneMapper are registered trademarks of Life Technologies Corporation.

2. STR Profiling Results

STR Locus	Test Sample: T-47D		Reference Sample: T-47D breast cancer cells	
Amelogenin	X		X	
D3S1358	15	17	15	17
D1S1656	15	16	15	16
D2S441	14		14	
D10S1248	17		17	
D13S317	12		12	
Penta E	7	14	7	14
D16S539	10		10	
D18S51	17		17	
D2S1338	24		24	
CSF1PO	11	13	11	13
Penta D	10	12	10	12
TH01	6		6	
vWA	14		14	
D21S11	28	31	28	31
D7S820	11		11	
D5S818	12		12	
TPOX	11		11	
DYS391				
D8S1179	13		13	
D12S391	19		19	
D19S433	14.2		14	
FGA	23		23	
D22S1045	15		15	

Total alleles in the Test Sample **(a)**	10
Total alleles in the References Sample **(b)**	10
SHARED alleles, Test and Reference Samples **(c)**	10
Percent Match for Test and Reference Samples **(d)**	100%

NOTE:

i. The allele match algorithm compares the loci highlighted in grey only (8 core loci plus Amelogenin).

ii. Loci highlighted in grey (8 core STR loci plus Amelogenin) are used to verify cell identity using STR Database.

iii. Match Algorithm = $\dfrac{\text{SHARED ALLELES } \mathbf{(c)} \times 2}{\text{TOTAL ALLELES in the Test Sample } \mathbf{(a)} + \text{TOTAL ALLELES in the Reference Sample } \mathbf{(b)}}$

iv. Percent Match **(d)** = Match Algorithm x 100

3. Test Results

Refer (✓)	Comments
✓	The submitted sample profile showed **100% match** with the following reference cell line provided by customer.
	The submitted sample profile showed < 80% match for the ATCC human cell line(s) in the STR database. The submitted sample needs further testing for authentication of relatedness.
	The submitted sample profile is human, but not a match for any profile in the STR database.
	The submitted sample is a mixture. Multiple peaks are observed in fragment analysis results.

4. **Quality Control**
   Passed

5. **Test Laboratory Location**
   APICAL SCIENTIFIC Laboratory
   Apical Scientific Sdn. Bhd.
   No. 17, Jalan BS 7/1C,
   Taman Perindustrian Bukit Serdang,
   43300 Seri Kembangan,
   Selangor, Malaysia.

Report Issued by:
Che Nurul Fariza Che Hasnan (Laboratory Technician)

Report Authorized by:
Stanley Lim Soon Hong (Laboratory Officer)

This report shall not be reproduced except in full without approval of the laboratory. The result reported relates only to the test item received and tested in-house. The content of this report and any of the attachment is confidential to the recipient only.

End of Report
